# C/D box sRNA-guided 2′-*O*-methylation patterns of archaeal rRNA molecules

**DOI:** 10.1186/s12864-015-1839-z

**Published:** 2015-08-22

**Authors:** Patrick P. Dennis, Vanessa Tripp, Lauren Lui, Todd Lowe, Lennart Randau

**Affiliations:** Max-Planck-Institute for Terrestrial Microbiology, Karl-von-Frisch Strasse 10, 35043 Marburg, Germany; Department of Biomolecular Engineering, University of California, 1156 High Street, Santa Cruz, CA 95064 USA; Janelia Research Campus, Howard Hughes Medical Institute, 19700 Helix Dr, Ashburn, VA 20147 USA

**Keywords:** C/D box sRNA, Ribosomal RNA, RNA methylation, RNA-Seq, Hyperthermophiles

## Abstract

**Background:**

In archaea and eukaryotes, ribonucleoprotein complexes containing small C/D box s(no)RNAs use base pair complementarity to target specific sites within ribosomal RNA for 2'-*O*-ribose methylation. These modifications aid in the folding and stabilization of nascent rRNA molecules and their assembly into ribosomal particles. The genomes of hyperthermophilic archaea encode large numbers of C/D box sRNA genes, suggesting an increased necessity for rRNA stabilization at extreme growth temperatures.

**Results:**

We have identified the complete sets of C/D box sRNAs from seven archaea using RNA-Seq methodology. In total, 489 C/D box sRNAs were identified, each containing two guide regions. A combination of computational and manual analyses predicts 719 guide interactions with 16S and 23S rRNA molecules. This first pan-archaeal description of guide sequences identifies (i) modified rRNA nucleotides that are frequently conserved between species and (ii) regions within rRNA that are hotspots for 2'-*O*-methylation. Gene duplication, rearrangement, mutational drift and convergent evolution of sRNA genes and guide sequences were observed. In addition, several C/D box sRNAs were identified that use their two guides to target locations distant in the rRNA sequence but close in the secondary and tertiary structure. We propose that they act as RNA chaperones and facilitate complex folding events between distant sequences.

**Conclusions:**

This pan-archaeal analysis of C/D box sRNA guide regions identified conserved patterns of rRNA 2'-*O*-methylation in archaea. The interaction between the sRNP complexes and the nascent rRNA facilitates proper folding and the methyl modifications stabilize higher order rRNA structure within the assembled ribosome.

**Electronic supplementary material:**

The online version of this article (doi:10.1186/s12864-015-1839-z) contains supplementary material, which is available to authorized users.

## Background

Stable RNA molecules frequently undergo post-transcriptional modifications. The majority of these modifications involve the addition of methyl groups. The most prevalent modifications found in ribosomal RNA (rRNA) molecules are the methylation of the ribose moiety at the 2'‐hydroxyl group (2'-*O*-methylation) and the conversion of uridine to pseudouridine. In bacteria, 2'-*O*-methylation modifications are relatively rare and are introduced by dedicated site specific or region specific methyltransferases. In contrast, eukaryotes and archaea utilize ribonucleoprotein (RNP) complexes containing small (s)RNAs to identify targets for 2'-*O*-methylation. These sRNA molecules are called small nucleolar RNAs (C/D box snoRNA) in eukaryotes and the archaeal homologs are C/D box sno-like RNAs (C/D box sRNA). They are characterized by four conserved sequence elements termed box C and C' (consensus sequence: RUGAUGA) and box D and D' (consensus sequence: CUGA). These elements fold into a ubiquitous RNA structural motif, the kink-turn (k-turn) [1–3]. The standard k-turn structure consists of a short stem often containing non-canonical base pairs that is capped with two sheared base pairs (AG and GA) [3]. Typically C/D box sRNAs contain two k-turn motifs, the first generated through the interaction of the C and D box sequences at the 5' and 3' end of the molecule and the second generated from the D' and C' box sequences located in the center of the molecule. The conserved C and D' boxes (and C' and D boxes) flank short guide sequences typically 10 to 12 nts in length that are highly variable and frequently exhibit complementarity to sequences within rRNA [4]. In addition, the guide regions of some C/D box sRNAs exhibit complementarity to different tRNA molecules, whereas other guide regions appear to lack complementarity to either rRNA or tRNA [5].

In archaea, two copies of the L7Ae protein bind respectively to the C and D, and D' and C' box sequences and stabilize the k-turn structural motif. This is followed by the further addition of the proteins Nop5 and the fibrillarin methyltransferase to complete assembly of the active complex. The guide regions of the sRNA base pair with complementary sequences in the target RNA and mediate 2'-*O*-methylation to the nucleotide that is base paired with the guide five nts upstream from the start of the D or D' box sequences using S-adenosyl-methionine as methyl donor [4, 6–9]. The methyl group deposited at the 2'-OH moiety in the target RNA favours a 3' endo-conformation of the ribose and blocks sugar-edge interactions. In addition, stability against hydrolysis by bases and nucleases is increased [10, 11]. Thus, it is plausible that organisms growing at elevated temperatures require more 2'-*O*-methylated RNA nucleotides to support RNA folding and stabilization [5, 12].

Single-particle electron microscopy studies and the crystallization of archaeal C/D box sRNP complexes with different sRNA compositions revealed structures that either contained two or four copies of L7Ae, Nop5 and fibrillarin [13, 14]. Recent NMR spectroscopy experiments verified the presence of dimeric sRNP assemblies, each containing two C/D box sRNAs and four copies of each protein [8]. These studies revealed the potential for sequential methylation of target RNAs by the same dimeric C/D box sRNA complex. Thus, the presence of two guide sequences in each C/D box sRNA could have evolved to provide a regulatory mechanism for rRNA folding.

Previous studies along with our results indicate that C/D box sRNA genes and their transcripts are highly abundant in hyperthermophilic archaea [15, 16]. Initial studies to map methylation targets of archaeal C/D box sRNAs focused on small RNAs that were co-immunoprecipitated with fibrillarin and Nop5 (also called Nop56) from *Sulfolobus acidocaldarius*, which led to the identification of 18 different C/D box sRNAs and their targets [4]. Physical methods were used to show extensive post-transcriptional modifications in helices 90–92 in domain V of the 23S rRNA in both *S. acidocaldarius* and *Haloarcula marismortui* [17]. The advent of RNA-Seq methodology has allowed for the global profiling of small RNAs from archaeal model organisms and has revealed a plethora of experimentally verified C/D box sRNA sequences [15, 16, 18]. In this study, we utilized seven RNA-Seq datasets to deduce C/D box sRNA-guided 2'-*O*-methylation patterns of archaeal rRNA molecules. This pan-archaeal analysis revealed methylation hotspots in functionally important rRNA regions. Furthermore, C/D box sRNAs were identified that are proposed to function as RNA chaperones assisting rRNA folding during ribosome assembly.

## Results and discussion

### Identification of C/D box sRNA genes

We utilized Illumina RNA-Seq methodology to obtain a global overview of the production and maturation of small RNA molecules for different archaeal species. We initially compiled these small RNA profiles to investigate several unusual tRNA and CRISPR processing pathways [15, 16, 19] but soon realized that C/D box sRNA genes were numerous in hyperthermophilic archaea and that their transcripts were highly abundant and easily detectable. Therefore, we initiated a comprehensive comparison of the RNA-Seq datasets. We used these data sets to generate a pan-archaeal overview of C/D box sRNA-guided rRNA methylation (Additional file 1). This study used the small RNA profiles generated from seven archaeal species, covering three different phyla and six different orders (Table 1). Included is *Nanoarchaeum equitans*, a hyperthermophilic archaeon with a highly compacted genome of only 490 kb size which harbors a small set of only 26 C/D box sRNA genes under genome reduction constraints. Six of the species are thermophilic, with optimal growth temperatures of approximately 75 °C and above. We identified all abundant sRNA molecules (< 100 nt) in the RNA-Seq datasets. These sequences were manually searched for the presence of GA/AG k-turn motifs to define C and D boxes (Additional file 2). The regions between annotated C/D' box motifs and C'/D box motifs represent potential guide regions that are used to direct the 2'-*O*-methylation activity of the C/D box sRNP complex. Methyl modification occurs at the nucleotide in rRNA that forms a Watson/Crick base pair five nucleotides upstream from the start of the D' or D box sequences within the region of extended complementarity. This is the “N plus five rule”. We used this extended complementarity and the “N plus five rule” to produce an extensive list of predictions of the positions of methyl modifications within 16S and 23S rRNAs for each of the seven species (Additional file 1).

**Table 1:** List of the organisms analyzed in this study

Organism	Phylum/Order	Growth temperature	GC content (%)	No. of C/D box sRNA genes	RNA-Seq dataset
*Nanoarchaeum equitans* (Neq)	Nanoarchaeota	80–100 °C	31.6	26	[15]
*Ignicoccus hospitalis* (Iho)	Crenarchaeota/Desulfurococcales	80–100 °C	56.5	128	[15]
*Methanococcus maripaludis C5* (Mma)	Euryarchaeota/Methanococcales	35–40 °C	33.0	7	[19]
*Methanopyrus kandleri* (Mka)	Euryarchaeota/ Methanopyrales	84–110 °C	61.2	127	[16]
*Pyrobaculum calidifontis* (Pca)	Crenarchaeota/Thermoproteales	90–100 °C	57.2	88	[36]
*Sulfolobus acidocaldarius* (Sac)	Crenarchaeota/Sulfolobales	67–80 °C	36.7	61	[4, 5] and unpublished data
*Thermoproteus tenax* (Tte)	Crenarchaeota/Thermoproteales	70–97 °C	55.1	52	[18]

The archaeal organisms and their respective abbreviations, phylogenetic classification, growth temperature, number of sRNA genes and database citations are indicated

### Distribution and conservation of predicted 2'-*O*-methylation sites in 16S and 23S rRNA across archaeal species

**Fig. 1:**
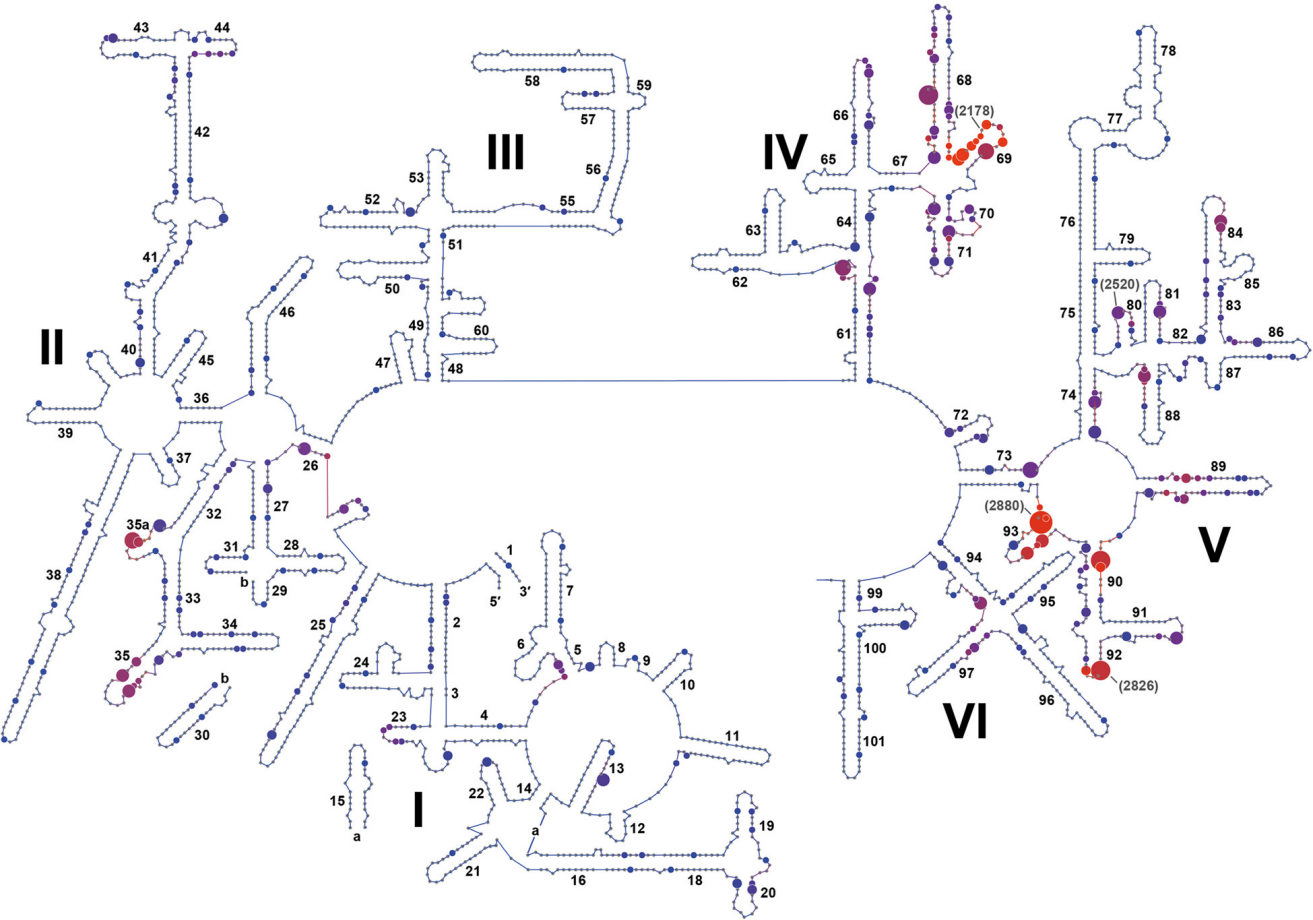
Distribution of 2′-*O*-ribose methylation sites in archaeal 23S rRNA. Predicted sites of sRNA directed 2′-*O*-ribose methylation (Additional Files 1 and 3) from seven archaeal species (listed in Table 1) were mapped onto the consensus secondary structure of the archaeal 23S rRNA [21]. The multiple occurrences of methylation at a given nucleotide position is indicated by increasing dot size (i.e. methylation targets found in one to six organisms). The color of the dots (from blue to red) indicates increasing methylation frequency within a nine nucleotide window. The archaeal nucleotide alignment positions of sites of modification discussed in the context of 23S rRNA structure-function are indicated

**Fig. 2:**
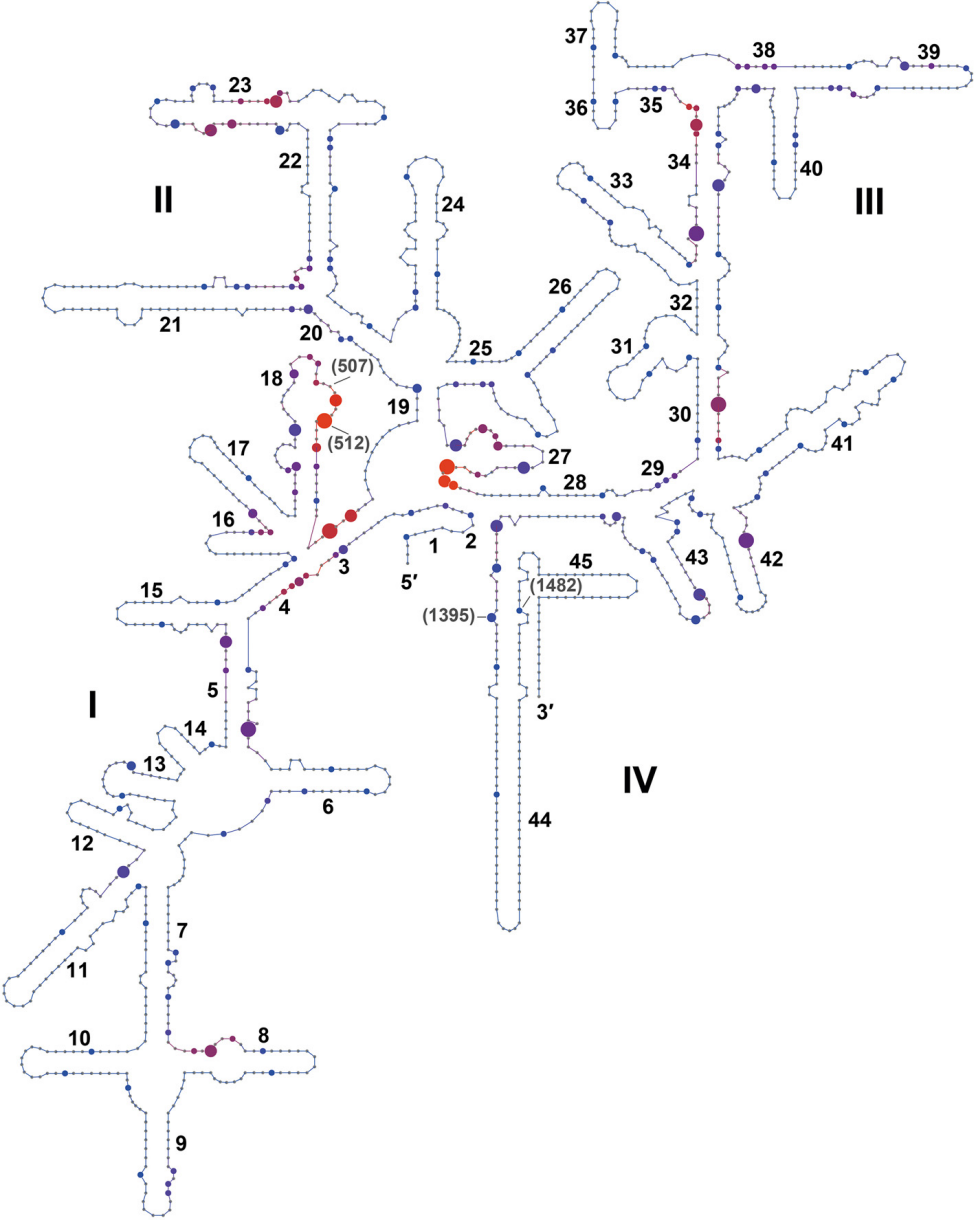
Distribution of 2′-*O*-ribose methylation sites in archaeal 16S rRNA. Predicted sites of sRNA directed 2′-*O*-ribose methylation (Additional Files 1 and 4) from seven archaeal species listed in Table 1 were mapped onto the consensus secondary structure of the archaeal 16S rRNA [21]. The multiple occurrences of methylation at a given nucleotide position is indicated by increasing dot size (i.e. methylation targets found in one to six organisms). The color of the dots (from blue to red) indicates increasing methylation frequency within a nine nucleotide window. The archaeal nucleotide alignment positions of sites of modification discussed in the context of 16S rRNA structure-function are indicated

To identify the distribution and conservation of sites of rRNA methylation, we first generated alignments of the respective 16S and 23S rRNA sequences from the seven divergent species of archaea (Additional files 3 and 4). The 719 predicted positions of modification (listed in Additional file 1) from the seven species were mapped onto the universal alignment and then located on secondary structure maps of 16S and 23S rRNA (Figs. 1 and 2). There were 266 predicted modifications in 16S rRNA that occur at 195 different positions and 453 predicted modifications in 23S rRNA that occur at 334 different positions. In 16S and 23S rRNA there are respectively 152 and 255 sites that are unique and modified in only a single species. In addition, there are 43 and 79 sites in the respective 16S and 23S rRNA sequences that are modified in two or more of the species.

### Methylation sites in 23S rRNA

The crystal structure of the *H. marismortui* large ribosomal subunit has been determined [20]. Since the primary sequence and secondary structures of the 23S rRNAs are highly conserved across the archaeal domain [21], we mapped predicted archaeal 23S rRNA 2'-*O*-methylation sites onto the archaeal consensus 23S rRNA. In the secondary structure, six domains extend from a central core. We identified hotspots of conserved methylation within helices 35 and 35a in domain II, 61 and 68 to 71 in domain IV, and 90 to 93 in domain V (Fig. 1). These are located in regions within the ancient core of the ribosome with domain V in the center of the large ribosomal subunit structure. A high number of tertiary interactions occur between domain V and domains IV and II. Domain III and a large part of domain I are less connected with other domains and therefore are believed to be later evolutionary additions [22]. They show a generally lower density of methylation targets. Clustering of 2'-*O*-methylations in the functional important and evolutionary conserved regions suggests that these modifications contribute to the folding, structural stabilization, assembly and function of the 23S rRNA within the large subunit of the ribosome.

The majority of hotspots (except for those in domain IV) are located in close proximity to the catalytic peptidyl transferase center (in domain V), where peptide bond formation and peptide release occurs [23]. All six thermophilic archaeal species displayed methylation of the 23S rRNA at alignment position U/C2880 at the base of helix 93 in domain V (corresponding to position C2606 in *Escherichia coli* 23S rRNA; Additional file 3). This position is commonly pseudouridylated in eukaryotes, while in bacteria and chloroplasts, pseudouridylation occurs one nucleotide upstream [24]. Helix 93 is part of the peptidyl transferase center and interacts with the CCA-tail of the P-site tRNA [23, 25]. The P-loop of helix 80 is also part of this ribosomal P-site. In *E. coli*, the 23S rRNA nucleotides 2251 and 2252 in helix 80 interact with the CCA-tail of the peptidyl-tRNA in the ribosomal P-site; post-transcriptional modifications at these positions are reported for several organisms [25–27]. In our data, we predict modification of position G2520 (i.e. *E. coli* G2251) in three organisms.

In general, the concentration of methylation sites near the peptidyl transferase center in archaea resembles the pattern of posttranscriptional modification of nucleotides (base methylations and pseudouridylations) in the 23S rRNA of *E. coli* [28]. A MALDI-MS analysis focusing on helices 90–92 in domain V of 23S rRNAs from different bacterial and archaeal species, identified numerous sites of modification but few of the sites were conserved between the different organisms [17]. The A-loop in helix 92 contacts the conserved CCA-end of aminoacyl-tRNA in the A-site of the ribosome and is a hotspot for methylation in all three domains of life [17, 29]. This is a further example for the importance of modifications at RNA-RNA interaction sites. In our data set the first nucleotide of the A-loop (helix 92, position U2526; *E. coli* U2552) is predicted to be modified in five of the species and the fifth nucleotide in the loop (position C2830; *E. coli* C2556) is predicted to be modified in two species. For *S. acidocaldarius,* our predicted sites of modification agree with the methylation sites reported previously [17]. In the mesophilic species *Methanococcus maripaludis* only a few C/D box sRNAs exist and in this species the A-loop does not appear to contain any sites of guide directed methyl modification.

Helices 68, 69 and 71 in Domain IV are 2'-*O*-methylation hotspots but are not in direct proximity to the peptidyl transferase center. However, these helices are part of the interface between the large and the small ribosomal subunit [30]. The *E. coli* ribosome crystal structure revealed an interaction between A1912 in 23S helix 69 and C1407 and G1494 in 16S helix 44. Conserved pseudouridylation sites were shown to exist in helix 44 and the loop region of helix 69; these modifications influence RNA folding and are crucial for ribosome subunit association [24, 31]. In hyperthermophilic archaea, there is a high density of predicted 2'-*O*-methylation sites in helix 69 around *E. coli* position A1912 (position A2178 in our alignment). In 16S helix 44 methylation predictions are relatively sparse (Fig. 2); nonetheless predictions occur at A/G1395 (*E. coli* position A1408 adjacent to C1407) in two species (*S.acidocaldarius* and *N. equitans*) and at G1482 (*E. coli* position G1494) in the mesophilic species *M. maripaludis*.

Most of the RNA in the large subunit of the ribosome is protected and stabilized by the presence and binding of ribosomal proteins. The proteins often contain elongated termini that penetrate between RNA helices, interacting with several RNA domains to stabilize tertiary RNA structure [20]. The exceptions to this, where no ribosomal proteins are found, are the 23S regions of rRNA that forms the interface with the 30S subunit (involving helices 67–71 in domain IV and helices 32–35a in domain II) and the peptidyl transferase center (domain V). Our data indicate that these unprotected regions of 23S rRNA contain a higher density of methyl modification and that this likely helps stabilize secondary and higher order structure associated with subunit interaction.

### Methylation sites in 16S rRNA

Next, we analyzed the locations of conserved methylation sites within the 16S rRNA structure (Fig. 2). The four domains of the 16S rRNA are connected by a central core that is located in the neck region close to the functional decoding center of the small ribosomal subunit that is formed in the early stage of assembly [32]. This region containing helices 3, 18 and 27, also exhibits the highest density of conserved 2'-*O*-methylation target site predictions. The methylated nucleotides in this region likely contribute to the stabilization of the decoding center as well as the tight association of the four domains.

Helix 18 (along with helix 44) monitors the correct codon-anticodon pairing and is the core of the decoding center in the small ribosome subunit [33, 34]. The G507 (G530 in *E. coli* 16S rRNA) is intimately associated with the interaction of the A site tRNA anticodon with the mRNA codon; site directed mutations at this position are lethal [35]. Other mutations in this region affect translational fidelity and resistance to the antibiotic streptomycin.

In five of the seven species examined there are a total of 23 methylation predictions at 13 different positions within helix 18. Seven of the 13 sites are modified in only a single species whereas six of the sites are modified in two or more of the species. Positions A509 and A512 (adjacent to G507; *E. coli* position G530) are predicted to be methylated, respectively, in three and four archaeal species. We predict that the high density of predicted modifications contributes to the folding, structural stabilization and translational fidelity function of this helix. It is interesting that there are no predicted modifications in this helix in either *M. maripaludis* (a mesophile) or in *N. equitans* (organism with contracted genome).

### C/D box sRNAs that target conserved methylation sites

**Fig. 3:**
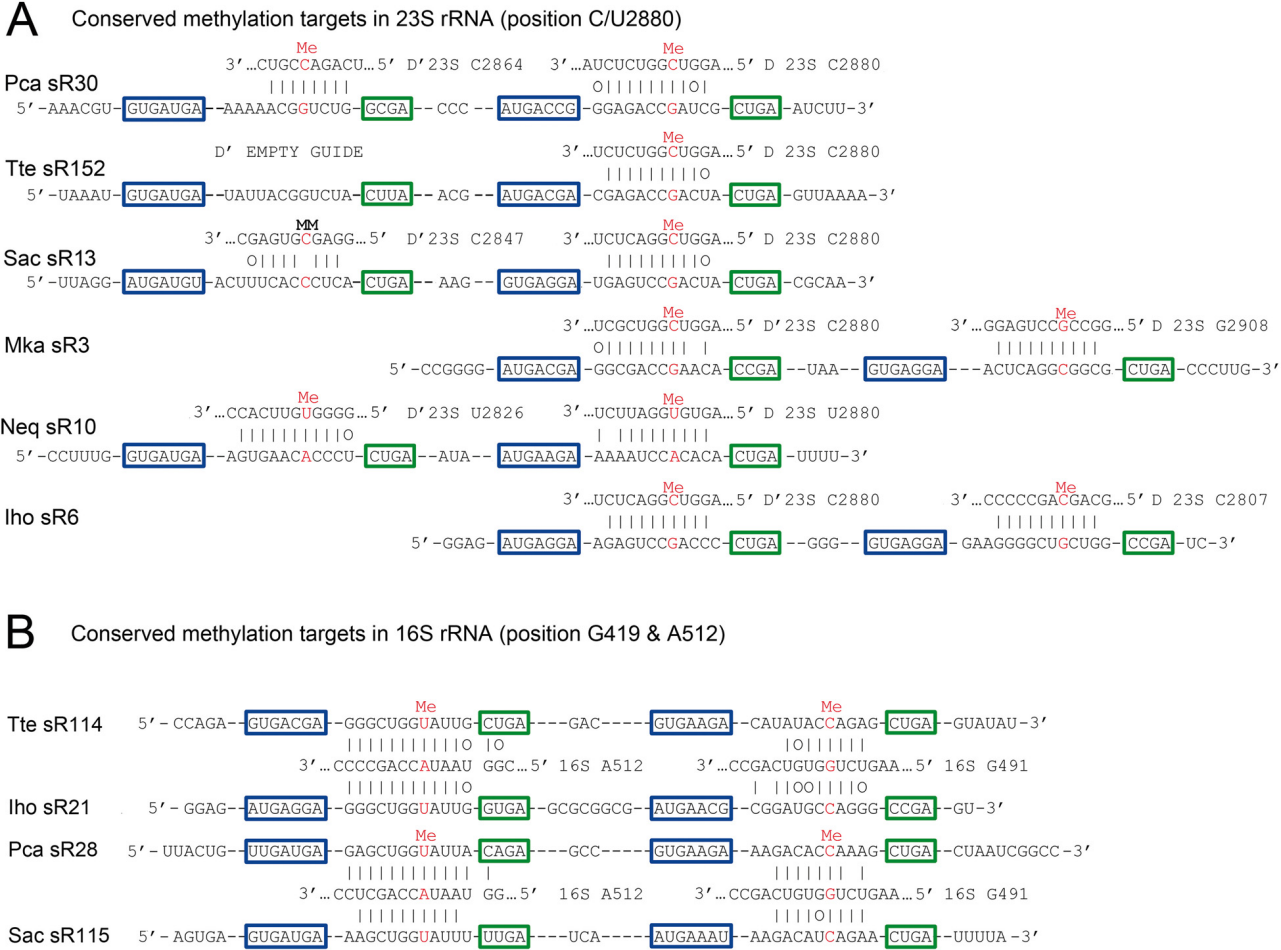
Complexes between sRNA guides and targets at sites of conserved 2′-*O*-ribose methylation in rRNA. **a** Conserved methyl modification of position 23S rRNA C/U2880. The complementarities between the D' and D guide regions of Pca sR30, Tte sR152, Sac sR13, Mka sR3, Neq sR10 and Iho sR6 with the 23S rRNA region around position C/U2880 (Eco position C2606; see Additional file 3) are illustrated with position 2880 aligned in the middle and labelled in red. The sRNA sequences for each species are listed below the rRNA sequences with the C and C' boxes outlined with blue and the D' and D box sequences outlined with green. The predicted positions of methyl modification are indicated by “Me” in red within the rRNA sequences. The D' guide of Tte sR112 has no significant complementarity to rRNA (empty guide). The D' guide-target complementarity of Sac sR13 exhibits a mismatch basepair at the predicted site of modification (C2847; MM:mismatch) and is likely not modified. **b** Conserved methyl modifications at positions 16S A512 and G491. The complementarities between the D' and D guide regions of Tte sR114, Iho sR21, Pca sR28 and Sac sR115 to the regions around 16S A512 (left of center) and G491 (right of center) are illustrated. These modification sites correspond to positions A535 and G515 in helix 18 of Eco 16S rRNA (Additional file 3). The sRNA C and C', and D' and D boxes are outlined with blue and green respectively. The predicted sites of methyl modification are indicated by (Me) in red. The sequence complementarity between the D guide of Iho sR21 and the region of around 16S G491 is disrupted by three GU base pairs (0) which likely compromises methylation activity; this Iho sR21 site was not considered to be a robust prediction in our analysis

Our analysis indicates that about 17 % of the predicted sites of methylation in rRNA are modified in more than one species and that the average number of species modifying at these multiple hit sites is just less than three. Position U/C2880 in our pan-archaeal alignment (located at the base of helix 93; C2606 in *E. coli* 23S rRNA) is predicted to be modified in all six of the thermophilic archaeal species examined in this study (See “Methylation sites in 23S rRNA” above). This means that each species contains a sRNA with a conserved guide region that exhibits complementarity to the 23S rRNA regions surrounding position U/C2880 (Fig. 3a). In Tte sR152 the D box guide is responsible for directing modification to 23S U2880; the D' guide in this sRNA exhibits no significant complementarity to either 16S or 23S rRNA. In Sac the sR13 D box guide again directs methylation to 23S U2880; the D' guide exhibits significant complementarity to a nearby region but appears to be defective in methylation at position C2847 because of a mismatch base pair at the predicted site of modification. The other four sRNAs are conventional double guide sRNAs: Pca sR30 uses the D box guide to target methylation to position C2880 and the D' guide to target methylation to C2864; Mka sR3 uses the D' guide to target methylation to C2880 and the D guide to target methylation to G2908; Neq sR10 uses the D box guide to target methylation to U2880 and the D' guide to target methylation to U2826; Iho sR6 uses the D' guide to target methylation to C2880 and the D guide to target methylation to C2807.

A more clear-cut example of sequence conservation between sRNAs from different species is represented by four sRNAs (Tte sR114, Iho sR21, Pca sR28 and Sac sR115) that use D' guides to direct modification to position 16S A512 (*E. coli* A535) (Fig. 3b) in helix 18 involved in mRNA-tRNA decoding (see above). The D guides of three of these sRNAs (Tte, Pca, and Sac) exhibit significant complementarity to a second closely located site and are predicted to direct modification to position G491. The D guide of Iho sR21 has a related D guide that contains three GU base pairs that partially disrupts this complementarity and it is uncertain if the sRNA complex is able form a complementary helix around G491.

There are numerous other examples of sRNAs from the various archaeal species that share a related guide sequence (see Figs. 1 and 2, Additional files 3 and 4 for sites that are modified in two or more of the seven species). The evolutionary origin of these related guide sequences is unclear. In the six sRNAs that target position 23S C/U2880, the shared guide is adjacent to the D box in four and adjacent to the D′ box in two cases (Fig. 3a). The other guides in the six sRNAs are unrelated in sequence. The second group of four sRNAs where both guides appear to target conserved sites is even more confounding (Fig. 3b). It is possible that the four sRNAs are either derived from a common ancestor or originated through sequence convergence. In either case there seems to be strong selection for the coupled interaction of the two guides with the 16S rRNA sequences surrounding the G491 and A512 sites of modification. In the numerous other instances of modification in two or more species, many are likely the result of evolutionary convergence.

### Diversification of C/D box sRNA guides within species

We have noted especially within Mka and Iho, that there are frequently two or more sRNAs that are predicted to target modification to the same site in rRNA (Table 2). These instances are of interest since they provide clues relating to the origin, propagation and diversification of sRNA genes. There are a number of possible mechanisms that can give rise to the observed redundancy including (i) sRNA gene duplication, (ii) recombination between sRNA genes, (iii) insertional mobilization of guide regions (along with the flanking C and D box sequences) and/or (iv) sequence convergence.

**Table 2:** List of C/D box sRNAs that target the same site within a species

Species	Subunit	Site	sRNAs
Mka	16S	C122	sR39/119
Mka	16S	G152	sR39/119
Mka	16S	C250	sR24/25
Mka	16S	G660	sR41/52/92
Mka	16S	G687	sR10/41
Mka	23S	G616	sR37/90
Mka	23S	G925	sR77/95
Mka	23S	G1237	sR21/29
Mka	23S	G1710	sR55/92
Iho	23S	G2140	sR314/96
Mka	23S	G2151	sR77/95
Mka	23S	G2520	sR13/45
Mka	23S	G2761	sR56/103
Iho	23S	C2864	sR124/127

The genomes of Mka and Iho contain a large number of sRNA genes (see Table 1) and within these species there are instances where two or three sRNAs are predicted to target methylation to the same position in rRNA. In the other five species with fewer sRNA genes, there are no sRNAs that are predicted to target methylation to the same position

**Fig. 4:**
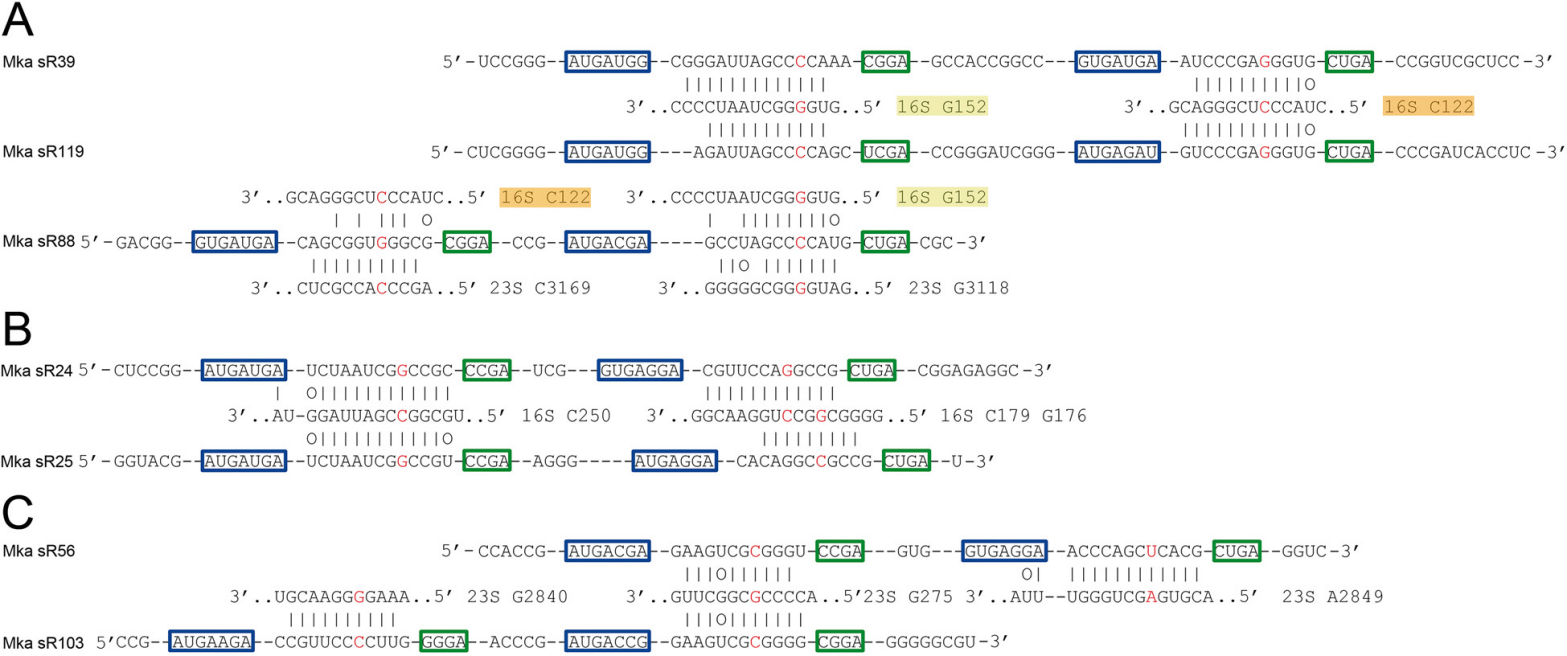
Sequence conservation and divergence within orthologous sRNA genes. The notation for box sequences and predicted positions of rRNA modification are described in the legend to Fig. 3. **a** The Mka sR39 and sR119 sRNAs are derived from duplicate genes and exhibit a high degree of end-to-end sequence similarity. The sR88 has a D box guide that exhibits high similarity to the D' guides of sR39/119 and a D' guide that exhibits only residual similarity to the D guide of sR39/119. The predicted D and D' target sites for sR88 are 23S G3118 and C3169; in our analysis, 16S G152 was not considered a D guide-target for sR88 because it did not meet the prediction criteria. **b** The Mka sR24 and 25 sequences are derived from duplicated genes. The 5' and 3' regions of the D guides are affected by indels that shift the site of modification between C179 and G176. **c** The Mka D' guide of sR56 and the D guide of sR103 are highly similar in sequence and are predicted to guide modification to 23S G2759. The D guide of sR56 and the D′ guide of sR103 are unrelated and predicted to guide modification to positions 23S A2849 and G2840 respectively

The Mka sR39 and sR119 are derived from duplicate genes that share a modest degree of sequence similarity in the immediately adjacent flanking sequences (Fig. 4a). The two sRNAs use their D and D' guides to direct modification of positions C122 and G152 in 16S rRNA. A third sRNA, Mka sR88 has a D guide that is similar to the D' guides in sR39 and 119 and may be capable of directing modification to position 16S G152. The D' guide of sR88 shows a low level of residual similarity to the sequence around 16S C122 but is highly unlikely to be active at this site. Thus it seems that sR88 represents a more divergent duplication of the sR39/119 family that has undergone a circular rearrangement resulting in a repositioning of the two guide sequences and a deterioration in the complementarity between the D' guide and the region surrounding the 16S C122 target. The most compelling modification predictions for sR88 are at 23S G3118 and C3169 (Additional file 1). A number of other scenarios involving sRNA gene recombination or guide mobilization can also explain the shared features of these three sRNA genes.

The Mka sR24 and 25 also appear to be derived from a gene duplication (Fig. 4b). The D' guides in the two sRNAs are virtually identical and are predicted to guide modification to position 16S C250. In contrast, the D guides contain a number of indels at the 5' and 3' ends of the guide regions that shift the predicted site of modification from 16S C179 for sR24 to G176 for sR25. Indels that occur at the 3' end of duplicate guides can explain the numerous instances where modifications are predicted at neighboring positions within 16S or 23S rRNAs.

A more complex example of target redundancy occurs with Mka sR56 and 103 where there is duplication and rearrangement of only one of the guide modules (Fig. 4c). As a consequence of these events the related D' guide of sR56 and the D guide of sR103 are predicted to target modification to position 23S G2759. The unrelated D guide of sR59 and the D' guide of sR103 are predicted to target modification to positions 23S A2849 and G2840 respectively. This example suggests that modules consisting of a guide sequence surrounded by C and D box sequences are fluid and can be either recombined or mobilized between two sRNA genes. After a gene duplication or rearrangement, processes generating nucleotide substitutions and indels in the guides can generate a substantial diversity for interactions involving sRNA guide sequences and rRNA target sequences.

### C/D box sRNAs as RNA chaperones

There are two consequences of the interaction of double guide C/D box sRNAs with two closely positioned sequences within rRNA. The first is the methylation of the 2'-*O*-ribose position in the RNA backbone that contributes to added stability in secondary and tertiary structure. Less apparent is the role that the dual interactions play in facilitating the localized folding events within the assembling ribosomal subunits. Many of the more critical and functionally important regions of rRNA are hotspots for guide-target interactions even though the exact positions of methyl modifications are not always conserved (see Figs. 1 and 2).

**Fig. 5:**
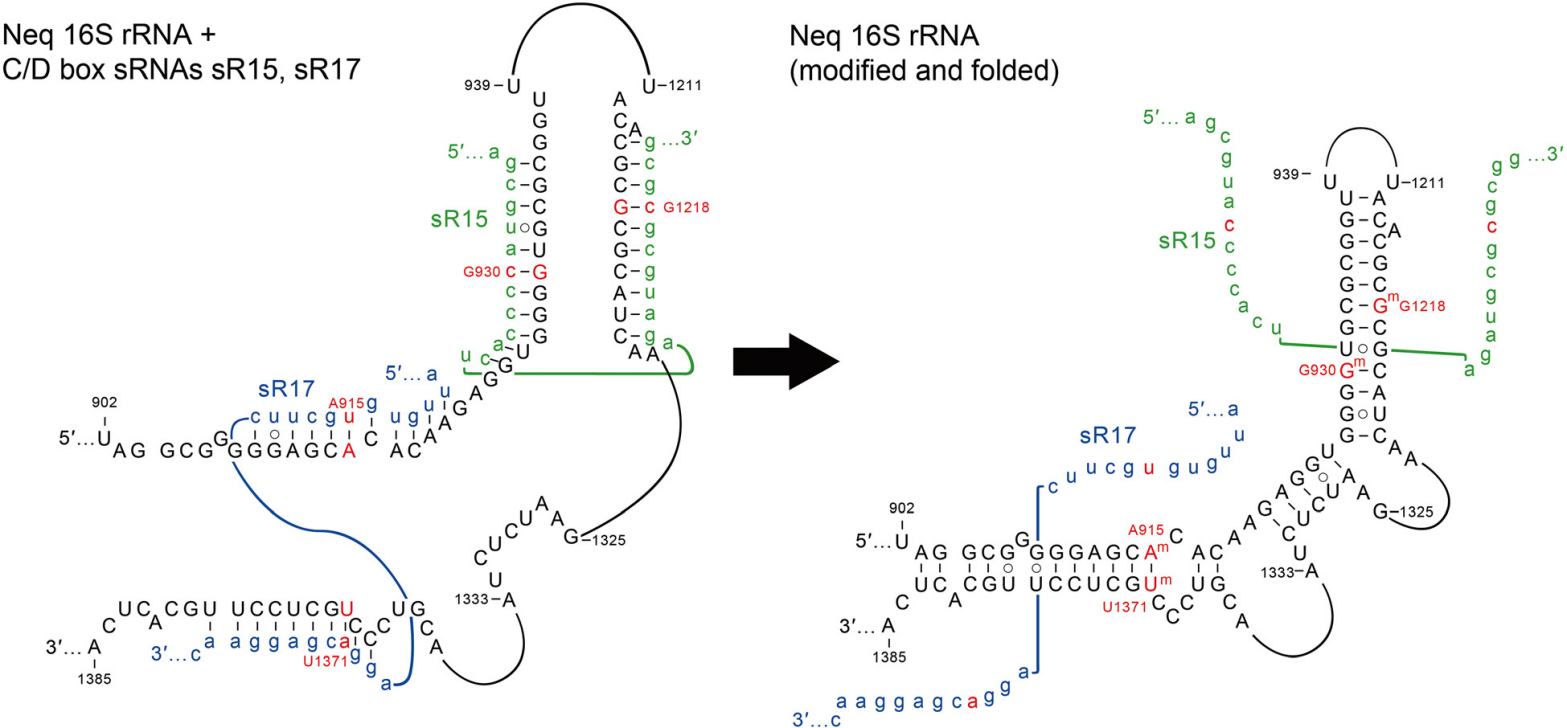
A chaperone function for C/D box sRNAs in long distant 16S rRNA folding. In *Nanoarchaeum equitans*, the guide regions of sR17 and sR15 exhibit extended complementary to the two strands of helix 28 and 30 of 16S rRNA respectively. The sRNA-rRNA interactions (*left*) position the distant rRNA sequences into close proximity. Release of the sRNAs following methylation at positions A915, G930 G1218 and U1371 is postulated to play a critical role in the efficient folding of helices 28, 29 and 30 in the nascent 16S rRNA (*right*)

Because of the obvious role of sRNA-rRNA guide-target interactions in localized rRNA folding, we wondered if such interactions might also occur at more distant locations to facilitate complex folding events. We therefore looked at sRNAs that exhibit complementarity to widely separated positions within the primary rRNA sequences. We found a number of intriguing interactions (Table 3). The most interesting of these occurs in *N. equitans* and involves the interactions of sR17 and sR15 with 16S rRNA (Fig. 5). The first interaction involves pairing of the D and D' guides of sR17 with the sequences around A915 and U1371 that are located on opposite strands of what will become helix 28. The second interaction involves similar pairing of the D and D' guides of sR15 with the rRNA sequences around positions G930 and G1218 that are located on opposite strands of what will become helix 30. The sR17 interaction sequesters each of the strands of the basal helix that defines domain III of 16S rRNA. The second interaction sequesters the opposite strands of the nearby helix that defines a large subsection of domain III. We suggest that these guide-target interactions play an important role in bringing the distantly separated 16S rRNA sequences into close proximity and facilitating their formation into helices following the target methylation and release of the sRNAs. A number of other potential long-range guide-target interactions that may play an important role in ribosome subunit assembly have been identified (Table 3). In addition, we suggest that the dimeric C/D box RNP complexes observed in NMR spectroscopy experiments [8] may use their quadruple guides to target sites that are distant in the primary rRNA sequence; these distant interactions could represent a complex and important mechanism for sequestering distant rRNA sequences and bringing them into close proximity within the assembling ribosome in a manner analogous to what is predicted for Neq sR15 and 17 (Fig. 5).

**Table 3:** List of C/D box sRNAs that target distant sites that are close in secondary structure space

Species	sRNA	Subunit	D target	D' target
Iho	sR114	23S	C1961	C2265
Iho	sR114	23S	C2275	D1937
Iho	sR472	23S	C897	G1017
Iho	sR206	16S	G*36 and 505	G482
Iho	sR103	16S	C1045	U1136
Pca	sR53	16S	A509	C34 and 530
Pca	sR56	16S	U894	G1358 and G925
Pca	sR2	23S	G747	C884 and 769
Mka	sR87	23S	A1241	G1385
Neq	sR15	16S	G1217	G930
Neq	sR17	16S	U1370	A915
Tte	sR51	23S	C1947	G2249 and 1980
Tte	sR50	23S	U2883 and 2783	U2753
Tte	sR46	16S	U547 and 984	G924
Tte	sR15	16S	A509	C35 and 530

Five species (Iho, Pca, Mka, Neq, and Tte) have sRNAs that are predicted to target methylation to sites that are distant in the primary rRNA sequence but close in the secondary structure. Two types of interactions are observed. In the first, the D and D′ guides have single targets as exemplified by Neq sR15. In the second, one guide has a single target, whereas the second guide has two targets, one linked and one distant in the primary sequence but close in the secondary structure. An example of this is Tte 509 where the D guide is predicted to modify position 16S A509 and the D′ guide is predicted to modify at the linked position C530 and the distant position C35. In one instance, the Iho sR206 interaction with the distant site at position 16S G36 contains a mismatch base pair at the site of methylation and is predicted not to be modified although the guide-target may well occur. The asterisk (*) indicates a mismatch base pair at the target site in the region of guide-target complementarity

## Conclusions

The pan-archaeal analysis of experimentally verified C/D box sRNA molecules was used to deduce 2'-*O*-methylation patterns for archaeal rRNA molecules. Hot-spots for modifications were found to be located in core regions of the ribosome, at the interface between the small and large ribosomal subunits and at sites of RNA-RNA interaction. These observations are in agreement with an increased need for 2'-*O*-methylated nucleotides in rRNA regions that lack protein protection to stabilize these sequences at high temperatures. The analyses of guide sequences revealed the accelerated evolution of C/D box sRNA genes with instances of gene duplication, rearrangement and guide sequence diversification. Moreover, many conserved target sites across the archaeal kingdom hint at the convergent evolution of the corresponding guide sequences. The presence of two guide sequences in each C/D box sRNA allows for the sequential methylation of two target sites. Our analysis of guide-target interactions suggests that the two potential targets of an sRNA are often closely linked (within 100 nts in the primary rRNA sequence) and the simultaneous interactions likely play an important role in facilitation rRNA folding events. Similarly, we identified C/D box sRNAs with targets separated by more than 100 nts that are required to be brought in close proximity during rRNA folding. These distant interactions provide further evidence for a potential RNA chaperone function of C/D box sRNAs.

It should also be noted that RNA-guided methylation adds to ribosome heterogeneity as the methylation of individual nucleotides is not always absolute. Elevated growth temperatures could result in altered target recognition which would increase ribosome heterogeneity and represent another level of adaption to extreme growth conditions. Future analysis of the methylation status of all individual rRNA nucleotides and the abundance of various sRNAs under different physiological conditions will be necessary to evaluate the importance of site specific methylation and the degree of rRNA heterogeneity.

## Methods

### RNA-Sequencing

RNA extraction was performed on log-phase cells using the mirVana miRNA isolation kit (Ambion) according to the manufacturer's instructions. Illumina Hiseq 2000 RNA-Seq methodology was applied as described before [15, 16]. RNA-Seq and cDNA library preparation of *P. calidifontis* small RNA was performed using a Roche/454 GS FLX sequencer as described [36]. The sampling conditions and RNA-Seq library preparation protocols are detailed for each organism in the references provided in Table 1.

### Identification of small RNA species

Illumina TruSeq sequencing reads with a quality score limit of 0.05 were trimmed to remove linkers and poly-A tails and mapped to the reference genomes (GenBank: Neq NC_005213; Iho NC_009776; Mma NC_009135; Mka NC_003551; Sac NC_007181, Tte NC_016070) with CLC Genomics Workbench 5.0 (CLC Bio, Aarhus, Denmark). The following mapping parameters were employed (mismatch cost: 2, insertion cost: 3, deletion cost: 3, length fraction: 0.5, similarity: 0.8). Reads below 15 nts were not considered. The program was also used to determine the coverage of individual RNA molecules. All predicted RNAs and their 5' and 3' termini were manually verified and all intergenic regions were checked for the presence of RNAs with sequence coverage of less than 1000 reads. Gene annotations (including tRNA genes) were obtained from Genbank [37].

### Prediction of C/D box sRNA guide directed 2'-*O*-methyl modification of ribose residues in ribosomal RNA

Guide directed 2'-*O*-ribose methyl modification occurs in the target rRNA nucleotide that is base paired with the nucleotide in the guide regions of the C/D box sRNA that is located at the plus five position upstream from the start of either the D or D' box sequences. Methylation sites in rRNA were computationally predicted by scanning for extended complementarity between the D and D' guide regions of the sRNAs and corresponding species-specific 16S and 23S rRNA sequences. One mismatch, two GU base pairs, and no bulges were allowed in the duplex between the guide and the rRNA region containing the target nucleotide. The first nucleotide of the D or D' box was included in the guide sequence, as this position may participate in the formed duplex [5]. From the set of target candidates, predictions of methylation sites were manually curated and mapped onto the rRNA alignment. In a small number of instances within the 489 C/D box sRNAs examined in this study, there was considerable uncertainty regarding the identification and exact positioning of the D or D' box because of divergence from the normal spacing and from the CUGA consensus; in these few instances we made no attempt to predict targets. Where we were able to predict D and D' box sequences with reasonable certainty based on positioning and similarity to the consensus, predictions were generally considered significant if the guide-target complementarity contained at least nine consecutive canonical Watson-Crick base pairs and encompassed the plus five nucleotide methylation site (for exceptions to this general rule, see below). Of the 489 sRNAs examined approximately, one-third (163/489) failed to exhibit significant guide complementarity to either 16S or 23S rRNA; however, many of these sRNAs did exhibit complementarity of tRNAs or 5S rRNA (data not compiled).

Frequently C/D box sRNAs utilize both the D and D' guides to direct modification to separate targets within a region of 100 nucleotides in the rRNA sequence; these are called “double guide” sRNAs. In a small number of instances, the nine base pair complementarity criteria for target prediction was relaxed somewhat for these double guide sRNAs by allowing G:U base pairs and/or a single nucleotide mismatch base pair in the region of complementarity. The rationale for this was that simultaneous interaction of the two sRNA guides with adjacent sequences within rRNA results in thermodynamically more stable structure than a single guide-target interaction. Of the 489 sRNAs examined, approximately half (262/489) were predicted to function as double guide sRNAs. In a small number (16/262) of the double guide sRNAs, we noted that one of the two guide-target complementarities contained a mismatch base pair at the predicted site of modification. In spite of the mismatch, we believe that both guide-target interactions form in the complex but that methylation occurs only at the single position where the critical WC base pair is present [38, 39].

From the collection of 489 sRNAs, each containing two guide regions, we identified a total of 735 guide interactions that satisfied the above significance criteria. We predict that 719 of these interactions result in methylation of the target rRNA whereas 16 of the interactions are likely non-productive because of the lack of the critical WC base pair in the guide-target interaction.

## Data availability

RNA-Seq data are available at NCBI's Gene Expression Omnibus (GEO) database as series GSE44979 (*M. kandleri*) and GSE38821 (*N. equitans*, *I. hospitalis*).

Sequencing reads of *P. calidifontis* are provided at the UCSC Archaeal Genome Browser (http://archaea.ucsc.edu) [40]. C/D box sRNA sequences, abundance and target predictions are included within the article (and its additional files).

## Additional files

The following additional data are available with the online version of this paper.

Additional file 1 is an Excel file with two sheets. The first sheet lists the 489 sRNAs from the seven species listed in Table 1. The second sheet contains the list of predicted sites of 2′-*O*-methylation in 16 and 23S rRNA for the D and D′ guides of each sRNA.

Additional file 2 is a figure that details the conservation of C/C′ and D/D′ box sequences using sequence logos.

Additional file 3 is an alignment of 23S rRNA sequences from *E. coli* and the seven archaeal species listed in Table 1. The predicted sites of sRNA mediated methylation for each archaeal species are indicated in the alignment.

Additional file 4 is an alignment of 16S rRNA sequences from *E. coli* and the seven archaeal species listed in Table 1. The predicted sites of sRNA mediated methylation for each archaeal species are indicated in the alignment.

## Additional files

Additional file 1:
**Identified archaeal C/D box sRNAs and predicted rRNA target sites.** (Sheet 1) The identified C/D box sRNAs are listed for each of the seven archaeal species along with their genomic coordinates and orientation. The box C/C′ and box D/D′ sequences are in red and green respectively. (Sheet 2) The predicted sites of 2′-O-ribose methylation in 16S and 23S rRNA for the sRNAs are listed. Predictions are based on the criteria stated in the methods section. Some guides are predicted to have more than one target. Guides that did not meet the prediction criteria are indicated by NP (No Prediction); many of these have predicted targets in tRNAs or 5S rRNA that are not catalogued. Predictions followed by an asterisk (*) indicate that there is a mismatch base pair at the target site in the region of guide-target complementarity; in these instances the second guide in the sRNA has a predicted target that is within 100 nts of the mismatch site. (XLSX 104 kb) Additional file 2:
**Conservation of C and D boxes. The conservation of C/C′ and D/D′ box sequences is detailed using sequence logos.** The overall height of the stacks of nucleic acid symbols indicates sequence conservation and the height of symbols within the stack indicates the relative frequency of each nucleic acid at that position [41]. (EPS 4363 kb) Additional file 3:
**Methylation predictions in 23S rRNA alignment.** The 23S rRNA sequences from the seven archaeal species used in this study were aligned using Infernal with manual adjustments; a consensus sequence (CON) is given at the bottom. The 23S rRNA sequence from *Escherichia coli* was included in the alignment for reference with positions numbered separately. Highlighted bases in the alignment are as follows: (green) D guide predictions; (blue) D′ guide predictions; (magenta) sites that are predicted by more than one sRNA guide; (red) predictions that contain a mismatch base pair at the site of modification in the region of guide-target complementarity. The sRNAs responsible for each prediction are listed above the highlighted bases (shaded in grey) in the alignment; species abbreviations for sRNA designations are (S) Sac; (P) Pca; (K) Mka; (M) Mma, (N) Neq; (I) Iho; (T)Tte. (DOCX 80 kb) Additonal file 4:
**Methylation predictions in 16S rRNA alignment.** The 16S rRNA sequences from the seven archaeal species used in this study were aligned using Infernal with manual adjustments; a consensus sequence (CON) is given at the bottom. The 16S rRNA sequence from *Escherichia coli* was included in the alignment for reference with positions numbered separately. Highlights and abbreviations are as listed in the legend to Additional file 3. (DOCX 57 kb)

## Abbreviations

snoRNA: Small nucleolar RNA
sRNA: Small RNA
rRNA: Ribosomal RNA
k-turn: Kink-turn
