# Supplementary material for: C/D box sRNA-guided 2′-O-methylation patterns of archaeal rRNA molecules
Source: BMC Genomics. 2015 Aug 22;16:632. doi: 10.1186/s12864-015-1839-z (PMC4644070; doi:10.1186/s12864-015-1839-z)
Supplement: Additional file 3: — Methylation predictions in 23S rRNA alignment. The 23S rRNA sequences from the seven archaeal species used in this study were aligned using Infernal with manual adjustments; a consensus sequence (CON) is given at the bottom. The 23S rRNA sequence from Escherichia coli was included in the alignment for reference with positions numbered separately. Highlighted bases in the alignment are as follows: (green) D guide predictions; (blue) D′ guide predictions; (magenta) sites that are predicted by more than one sRNA guide; (red) predictions that contain a mismatch base pair at the site of modification in the region of guide-target complementarity. The sRNAs responsible for each prediction are listed above the highlighted bases (shaded in grey) in the alignment; species abbreviations for sRNA designations are (S) Sac; (P) Pca; (K) Mka; (M) Mma, (N) Neq; (I) Iho; (T)Tte. (DOCX 80 kb) [file 12864_2015_1839_MOESM3_ESM.docx]

**23SrRNA alignment**

10 20 30 40 50 60 70 80 90

Eco GGUUAAGC-GACUAAGCGUACACGGUGGAUGCCCUGGCAGUCAGAGGCGAUGAAGGACGUGCUAAUCUGCGAUAAGCGUCGGUAAGGUGAUAUGAACCG

| | | | | | | | | 100

P116 P15

K54I131 K28 I96K54I449 K107

Pca CGCUCCGCA-CGGCAAAGCCGCCCGGUGGAUGGCUCGGCUCGGG-CGCCGAAGAAGGGCGUGGCAAGCUGCGAUAAGCCCGGGGUAGCCGCAGGCAGGCU

Mma UAGAUUUAU-CUAUUACCCUAUCUGGGGAAUGGCUUGGCUUGGAACGCCGAUGAAGGACGUGGUAAGCUGCGAUAAGCCCAGGCGAGACGCAUACAGUCA

Tte CCGUCCGCA-CGGUCAAGCCGCCCGGUGGAUGGCUCGGCUCGGG-CGCCGAGGAAGGGCGUGGCAAGCUGCGAUAAGCCCGGGGUAGCCGCAAGCGGGCG

Sac CUACCCAGG-GGCCGAAGCCUCCCGGUGGAUGGCUCGGCUCGGG-CACCGAAGAAGGGCGCGGCAAGCAGCGAAAUGCUCGGGUGAGGCGCAAGCAGCCG

Mka CCGCCGGGU-CGGCUAAGCCGUCGGGUGGAUGCCUUGGCUCGGG-CGCCGAGGAAGGCCGUGGCAAGCUGCGAUAAGCCCGGGCGAGGCGCAUGCAGCCG

Neq UCCGCAGGACCGCUGACCCCGCCCGGUGGAUGCCUGGGCUCGGG-AGCCGAAGAAGGGCGUGGCAAGCUGCGAUAAGCCAGGGGGAGGCGCACGCAGCCG

Iho GGGGCAACGGCGCGUAAGCCGCCCGGUGGAUGGCUCGGCUCGGG-CGCCGACGAAGGGCGUGGCAAGCUGCGAUAAGCCCGGGGGAGGCGCACGCAGCCG

CON ccgcccgca-cgccuAagCcgcCcGGuGgAUGgCUcGGCUcGGg-cgCCGAaGAAGGgCGuGGcAAGCuGCGAuAaGCccgGGggAGgCGCAagCaGcCg

100 110 120 130 140 150 160 170

Eco UUAUAACCGGCGAUUUCCGAAUGGGGAAACCCAGUGU----------------------GUUUCGACACACUAUCAUUAACUGAAUCCAUAGGUUA----
 | | | | | | | | | 200

P116

I96 K107 I58 K99 K76 K13K70K90

Pca UAGAACCCGGGAUCCCCGAAUGGGGCUUCCUGCCGGG-GCCGAAUAG--GCCCCGGCGCCCC------------------GU-AA---------------

Mma UUGAACCUGGGAUUUCUGAAUGGGACUUCCUAC---------UUU----------GUAAUCC------------------GU-UAA--------------

Tte UUGAACCCGGGAUUCCCGAAUGGGGCUUCCUACCGGG-GCCGAACA--GGCUCCGGUGCCCC------------------GU-AA---------------

Sac UUGACCCCGAGGUCCCCUAAUGGGAUAUCCUGCCGG-----GUUU-------CCGGCGCUCCCGGUU--------------U-AU---------------

Mka UGGAACCCGGGAUCGCCGAAUGGGACCUCUCGCCGGG-GCCGAAA--GGU-CCCGGCACUCCGGGGGGAAACCCCGGGCCCUCGU-GGCCCGGGGGAGUA

Neq UUGAUCCCUGGAUCCCCGAAUGGGACCUCCUGCCCGGGGGUUAACC-----CCGGGCGCCCC------------------GAUAA---------------

Iho UCGAACCCGGGAUCCCCGAAUGGGACUUCCUGCCGGGGGC-GAACA--GCCCCCGGCGCGGG------------------GAGAA---------------

CON UuGAaCCcggGaUccCcgAAUGGGacuUCcugCCgGG-Gccgaaca---g-cCcGGcgcccc------------------gu-aa---------------

180 190 200 210 220 230 240 250 260

Eco -----AUGAGGCGAACCGGGGGAACUGAAACAUCUAAGUACCCCGAGGAAAAGAAAUCA-ACC-GAGAUUCCCCCAGUAGCGGCGAGCGAACGGGGAGCA
 | | | | | | | | | 300

P20

I128 K90I449 I83 I55

Pca -----GGGGCGGGAACGCGGGGAAAGGAAACAUCUUAGUACCCGCAGGAAGGGAAGCCA-ACA-GGGACCCCCUGAGUAGGGGCGACCGAAAGGGGGAUA

Mma -----GGAUUGGGAACGCGGGGGAUUGAAGCAUCUUAGUACCCGCAGGAAGAGAAAUCA-AUA-GAGAUUCCGUUAUUAGAGGCGAUUGAACGCGGAUCA

Tte -----GGGGCGGGAACGCGGGGAAAGGAAACAUCUUAGUACCCGCAGGAAGAGAAACCA-ACA-GGGAACCCCUGAGUAGGGGCGACCGAAAGGGGGAGA

Sac AACUGGGAGUGGGAACCCCCCGAACGGAAACAUCUUAGUAGGGGGAGGAAAAGAAAUCA-AUU-GAGAUCCCCUGAGUAGGGGCGACCGAAAGGGGGACA

Mka CCCCCGGAGGGGGAACCCGCCGAACUGAAACAUCUUAGUAGGCGGAGGAAAAGAAAGCA-ACACGCGAUGCCGUGAGUAGGGGCGACCGAAAACGGCAGA

Neq -----GGGGCGGGAACGCGGGGAAGUGAAACAUCUCAGUACCCGCAGGAAAAGAAAGAGAAAU-UCGAUGGCGGGAGUAGGGGCGACCGAAACCGCCGUA Iho -----CCCGCGGGAACCCCCCGAACGGAAACAUCUUAGUAGGGGGAGGAAGAGAAACCACCAC-GGGAUCCCCCGAGUAGGGGCGACCGAAAGGGGGGUA

CON -----ggagcGGGAACgCgggGaAcgGAAaCAUCUuAGUAcccGcAGGAAgaGAAacca-aca-ggGAuccCcugAgUAGgGGCGAccGAAaggGggauA

270 280 290

Eco GCCCA---------------------------------------------------GAGCC-------------------UGAAUC--AGUG---UGUGU
 | | | | | | | | | 400

K79 K36 K54 K71 K79*

Pca GCCCAAACCAAAU-CCUCGCGGGACAACCGUGGGGAGAUGUGG-G-GC--UUGGGCCCGGG----------C----------AACC-GCCG-GC--GGGC

Mma GAGCAAACUGAAU-CCC-----UUCG------GGGAGAUGUGGUG-UUA-UAGGGCCUUCU---------UU-------------UCGCCU--G--UUGA

Tte GCCCAAACCAAAU-CCUCACGGGAUGACCGUGGGGAGAUGUGG-UGUUG-U-GGGCUCGGG---------------------UACC-GCCG-GC--GGGC

Sac GCCCAAACUAAAC-CUGCCGAUGAUAAGUCGGUGGGGAUGUGG-UGUUA-CGACCUCUAGCCUGAGGU---UC-GA-CCUCG-GCU-UUCCUAA--CCUA

Mka GGGCAAACUGAAC-ACCCGCCCGAA-AGGGCGGGUGGAUGUGG-GGUUG-CAGGCCCGGGC--------CGCCUA-------UGUG-GCCG--UCUCCCC

Neq GCCCAAACUGAAU-CCCGUAGGGA-AACCUACGGGAGAUGUGG-G-GUUG-UUGGGGGUGGC---------G--G--------CUU-UCCC-UC--CCGC

Iho GCCCAAACCAA-UUCCCCACGGGAUAACCGUGGGGAGAUGAGG-G-GUGGAUGCCCCGCGGCUCCCCCU---C-GAGGGG-A-GGG-GCCCGAC--CCUC

CON GccCAAACuaAAu-ccccacgggauaAccguggGgaGAUGuGG-g-uug-uugggccgggg----------c--g--------gcu-gcCc-ac--ccgc

300 310 320 330 340 350 360 370

Eco GUU---AGUGGAAGCGUCUGGAAAGGCGCG--CGAUACAGGGUGACAGCCCCGUACACAAAAAUGCACAUGCUGU------GAGCUCGAUGAGUAGGGCG
 | | | | | | | | | 500

S13

K110 K24 K74K6 K5 K110K6 I404K74 I124 I76 K81I304

Pca GGU---AGCCGAAGUGGGCUGGAAUGCCCC-GCCGUAGAGGGUGAUAGCCCCGUAGGCGAAACCGCCCGUGGCGGAGU-CCCGG-GGUCCCGGAGUACCU

Mma GAA--AAGCUGAAGUUGACUGGAACGUCAC-ACUAUAGAGGGUGAAAGUCCCGUAAGCGUAAUCGAUUCAGGUU-----UGAAGU-GUCCCUGAGUACCG

Tte GGU---AGCCGAAGUGGGCUGGAAUGCCCC-GCCGUAGAGGGUGAUAGCCCCGUAGGCUAAACCGCCCGUGGCGGAGU-CCCGG-GAUCCCGGAGUACCC

Sac UCU---AGCCGAACUCCCCUGGAACGGGGG-GCCAUAGAGGGUGAAAGCCCCGUAGGCUAAAGAUAGGUGGAAAG-U-GGCUAG--A-GGUAGAGUACCA

Mka CGGAACGGCCGAAGUGCCCUGGAACGGGCC-GCCGGAGAGGGUGACAGCCCCGUAGGCC-GCCGGGGGACGGCCG-CUGGCCCG--G-GCCUGAGUACCG

Neq GGGA--ACCCGAACUCCGGUGGAAGUCCGGGGCCGUAGAGGGUGACAGCCCCGUAGGGGUAACCGCGGAGGGAGU-G-CCGCCA-C-UUCCAGAGUACCG

Iho GGU---ACCCGAACUCCGGUGGAAGUCCGGGGCCGUAGAGGGUGACAGCCCCGUAGGGGAAACCGAGGGGGGCCC-U-GCCGCG--G-GGAUGAGUACCA

CON ggu---agCcGAAgUccgcUGGAAcgcccc-gCcguAGAGGGUGAcAGcCCCGUAgGcgaaaccgaggggGgcgg-g-gccccg--gUcccuGAGUACCg

380 290 400 410 420 430 440 450 460 470

Eco GGACACGUGGUAUCCUGUCUGAAUAUGGGGGGACCAU-CCUCCAAGGCUAAAUACUCCUGACUGACCGAUAGUGAACCAGUACCGUGAGGGAAAGGCGAAA
 | | | | | | | | | 600

S28 P63 P49P15

I304 K5 I86 K37K90 K121

Pca CGGCUUAGUUUUGCCGGGGGAACGCGCCGGCCACUGGCCGGCAAGGCUAAGCACGUCCCGAGUCCGAUAGCGCACU-AGUACCGUGAGGGAAAGCUGAAA

Mma UGCGUUGGAUAUCGCGCGGGAAUUUGGGAGGCAUCAACUUCCAACUCUAAAUACGUUCCAAGACCGAUAGUGUACU-AGUACCGCGAGGGAAAGCUGAAA

Tte CGCCUUGGUUUUGGCGGGGGAAGCUGGCGGCCACUGGCCGCCAAGGCUAAGCACGUCCCGAGUCCGAUAGCGAACU-AGUACCGUGAGGGAAAGCUGAAA

Sac UCCCCUGGUUUGGGGGUGGGAAGUUAGGGGACACGUGCCUCUAAGGCUAAAUAUGUCCCGAGACCGAUAGCAAACUAAGUACCGUGAGGGAAAGCUGAAA

Mka UCGGUUGGAUAUCCGGCGGGAAUCUGGGGGACAUCGGCCCCCAACCCUAAAUACGUCCCGAGUCCGAUAGCGAACA-AGUACCGUGAGGGAAAGGUGAAA

Neq CGCCUCGGAUAUGGCGCGGGAAGCUGGGGGACACACGCCCCCAAGGCUAAAUACUCCCCGAGUCCGAUAGCGGACU-AGUAGGGUGACCGAAAGGUGAAA

Iho CGGCUUGGUAUUGCCGUGGGAAGCUGGGUGGCACCAGCAUCCAAGGCUAAACACGUCCCGAGACCGAUAGCGAACUAGGUACCGUGAGGGAAAGCUGAAA

CON cgccuugGuuuuggcGcGGGAAgcuggggGaCAccggCcuccAAggCUAAauAcgucCCgAGuCCGAUAGcgaACu-aGUAccGuGAggGAAAGcUGAAA

480 490 500 510 520 530 540

Eco AGAACCCCGGCGAGGGGAGUGAAAAAGAACCUGAAACCGUGUACGUACAAGCAGUGGGAGCACGC------------------------------UUAG-

| | | | | | | | | 700

N3

K110 K37/90 K2 I415 K121

Pca AGAACCCCGG-AAGGGGGGUGAAAA-GAGCCUGAAACCGGGCGGCUACAGU-GGGGCAGGCCCGAAAGGA-UGCCCCCUCCCGAAGGAAACCCCGGUGAC

Mma AGCACCUUUA-AUCGGGUGUGAAAA-GAGCCUGAAACCAGAUAGGUAUGGUAUGACACGGCCCCAAAGG--C-AACUAUAUUGAAGGAAACCGUCGCAAG

Tte AGCACCCCGG-AAGGGGGGUGAAAA-GAGCCUGAAACCGGGCGGCUACAGU-GGGGCGGGCCCCAAAGGA-UGCCCUCGCCCGAAGGAAACCCCGGUGAC

Sac AGAACCCCGGAAGGGGGAGUGCAAA-GAGCCUGAAACCGGGUGGUUAUACA-GGGUGUGGCUCGAAAGAAGUGAACCCUUCCGAAGGAAAGGGGCGCAAG

Mka AGAACCCCGG-AAGGGGAGUGAAAA-GAGCCUGAAACCCGACGGCGAUAGUCUGCCGGGGCCCGCAAGGGAUGAAGCCCCCCGAAGGAAACCCCGGUGAC

Neq GAGAACCCGUGGAAGGGAGUGAAAAAGAGCCUGAAACCGGGCGGGGAUAGUCAGGUGGGGCCGGUUAA-----GCCCCCGGGCAAGGAACCCCCGGCGAC

Iho AGCACCCCUGGC-GGGGGGUGAAAA-GAGCCUGAAACCGGGCGGUCACACA-GGGUGCGGCCCGCAAGGGAUGAAG-CCCCGGAAGGAAACCCGGGCGAC

CON agaAcCccgg-aagGGGaGUGaAAA-GAGCCUGAAACCgGgcgGcuAuagu-gGguggGGCccgaaAgga-uGaacccccccgAAGGAAaccccgGcgAc

550 560 570 580 590 600 610

Eco --------------------------GCGUGUGACUGCGUACCUUUUGUAUAAUGGGUCAGCGACUUAUAUUCUGUAGCAAGGUUAACC----GAAUA---
 | | | | | | | | | 800

S118 S123 P2 S123 S101 P2 P37

I405K70 K13K51K76 I203I401K32K2 I405 K32I203 I401

Pca -GGGGGAGUACGA-GGGAGGGGGUCCAGGGUCUGCCCUUACGUCUAGAAACACGGGCCGGGGAGUUCACGGCCGUGGCGAGCCUAAGGGGUUCAA---CC

Mma -GUGGCUGUACGA-AGUAUAGA-GCCAGGGUUGUGUCGUCCGUUUCGAAAAACGGGCCGGGGAGUAUAUUGUUGUGGCGAGCUUAAGAUCUUCAC---GA

Tte -GGGGGAGUACGA-GGGCGAGGGUCCAGGGUCCGCCCUUACGUCUAGAAACACGGGCCGGGGAGUUCACGGCCGUGGCGAGUCUAAGGGGUUUAA---CC

Sac -CCCUUAGUACGA-GGAAGGGCGAUCGGGGUCACGCCUUUCGUCUUGAAACACGGGCCGGGGAGUUCACAUCAGUGGCGAGCUUAAGGAGAUUAU---CU

Mka -GGGGGAGUACGA-GGGGGGCCGACCGGGGUCCCGGCGUUCGUUUUGAAACACGAGGCGGGGAGUCCGCGGCCGCGGCGAGUCUAAGGGGUUCAAA--CC

Neq GGGGUAGUACUU-GCCCGGGGGCUAGCCGGUUCCACCGUACGUUCGGGAGCACGUGCGGGGGAGUGUGCCCGAGUGGCGAGGUUAAGGGGAUAAA---CC

Iho -CGGGGAGUACGUUCGGGUGCAGACCGGGGUCGCACCUUUCGUCUUGAAACACGGGCCGGGGAGUGCACGGCCGUGGCGAGGCUAAGGGGUUCAC---CC

CON -gggggaguacga-gggagggggaccagGGUcccgcCuUaCGUcuuGaAacACGgGccGGGGAGUucacggccGuGGCGAGccUAAGggguUcAa---cc

620 630 640 650 660 670

Eco GGGGAGCCGAAGGGAAACCG-----------------------------------------AGUCUUAACUGGGCGUUAAGUUGCAGGGUAUAGACCCGAA
 | | | | | | | | | 900

S124 P56 P2 T24

I64 I70 K51 I70 I12 K23 I121 I55 I472I415

Pca CCGGAGGCGUAGGGAAACCGAC-AGCCCGCAGCGGGG----CA--ACCCGCGAGGGGCGGGGUCUUAAA-GGGCCCGUAGUCACGGCCGUGAGACCAGAA

Mma UCGUAGGCGUAGGGAAACCAACAAGUCCGCAAA------AUC-------UUGAGGGACGGGGUCUUAA--GGGCCCGGAGUCACAGCAAUAUGACCCGAA

Tte CCGUAGGCGCAGGGAAACCGAC-AGCCCGUAGCCGGU--UUGC--GCCGGUGAGGGGCGGGGUCCGAAA-GGGCCUGUAGCCACGGCCGUGAGACCAGAA

Sac CCGAAGGCAUAGGGAAACCAAG-UGCCCGCAGCCUAG--UUU---CUAGGCGAGGGGCAGGGUCUGUCA-GGGCCUGAAGCCACUGAUGUGAGGCUAGAA

Mka CCGAAGGCGUAGGGAAACCGACACGCCCGCAGCCCGGCCGUUAGGCCGGGCCAGGGGCGGGGUCCUAAUAGGGCCCGCAGCCGCGGCCGCGGGACCCGAA

Neq CCGAAGCCGUAGGGAAACCGAA-GGCCCGCAGCCC------UU--AUGGGCGAGGGGCCGCGUCCUAUA-CGGGCGUAAGUCACUCGGGCACGACCAGAA

Iho CCGUAACCGCAGGGAAACCGACGAGCCCGCAG-GGGC--UCAU-GCCC--CGAGGGGCGGGGUCCGCAA-GGGCCCGGAGUCACGGUCGUGCGACCCGAA

CON cCGaAggCguAGGGAAACCgAc-aGcCCGcAgccggg--uuua--cccggcgAGGGgCgGgGUCcuaaa-gGGcCcgaAGuCaCggccgugaGaCcaGAA

680 690 700 710 720 730 740 750 760 770

Eco ACCCGGUGAUCUAGCCAUGGGCAGGUUGAAGGUUGGGUAACACUAACUGGAGGACCGA--ACCGACUA-AUGUUGAAAAAUUAGCGGAUGACUUGUGGCU
 | | | | | | | | | 1000

T24 P56 S18 S18* S120 P59T37P9

K99 K77/95 K67 I55 K42I131 K68 K77 K98 K67I77 K118 K98 K42K68

Pca ACCGGGCGAUCUAGCCCUGGGCAGGGUGAAGCGGGGCGAAAGCCCCGUGGAGGCCCGA--AGGGGUUCUGAUGUGCAAAUCGUUCCCAUGACCUGGGGCU

Mma ACCGGGCGAUCUAGGCCGGGGCAAGGUGAAGUCCCUCAACUGAGGGAUGGAGGCCUGC--AGAGUUGUUGCCGUUCGAAGCACUCUUCUGACCUCGGUCU

Tte ACCGGGCGAUCUAGCCCUGGGCAGGGCGAAGCGGGGCGAAAGCCCCGUGGAGGCCCGAA-AGGG-UUCUGAUGUGCAAAUCGUUCCCAUGACCUGGGGCU

Sac ACCGGGCGAUCUAGUCCGGGGCAGGCUGAAGGUGGGGGAAACCCCACUGGAGGGCCGAAUAGGGGUUCUGACGUGCAAUUCGUUCCCUUGACCUCGGACU

Mka ACCGGCCGAUCUAGCCCGGGGCAGGGUGAAGCGGCCCUAAAGGGCCGUGGAGGCCCGCU-AGGGGUGCUGUAGUGCAAAACGCUCCCGUGACCCCGGGCU

Neq GCCCGGCGAUCUACCCCGGGGCAGGGUGAAGCGGGGUU-UUUCCCCGUGGAGGCCCGA--AGGGGUGGUG---GCCUAUCCCCUCCUCUGACCCCGGGGU

Iho ACCGGGCGAUCUAGGCGGGGGCAGGGUGAAGCCGGGCGAAAGCCCGGUGGAGGCCCGCA-AGGG-UUCUGACGUGCAAUUCGUUCCCCUGACCUCCGCCU

CON aCCgGgCGAUCUAgcCcgGGGCAgGguGAAGcggggcgAaagccccgUGGAGGcCcGaa-AGgGgUucUGacGugCaAauCguUCcccUGACCucgGgcU

780 790 800 810 820 830 840 850 860 870

Eco GGGGGUGAAAGGCCAAUCAAACCGGGAGAUAGCUGGUUCUCCCCGAAAGCUAUUUAGGUAGCGCCUCGUGAAUUCAU-CUCCGGGGGUAGAGC-ACUGUU
 | | | | | | | | | 1100

T37S120P59 P9

K44 I207I77K99 K73I472 K73

Pca AGGGGCAAAAGACCAAUCAAGCCCGGUGAUAGCUGGUUCCCCCCGAAGCGGGUCUCAGCCCGGCCUCCCCGGAGGCGGCCGGCGGGGUAGAGU-ACUGAU

Mma AGGGGUGAAAGGCCAAUCGAGCCCGGAGAUAGCUGGUUCCCUCCGAAGUGACUCUCAGGUCAGCCGGAGUUUAGAUAGUCGGCAAGGUAGAGC-ACUGAU

Tte AGGGGCAAAAGACCAACCAAGCCCGGUGAUAGCUGGUUCCCCCCGAAGCGGGUCCCAGCCCGGCCUCCCUGGAGGUCUCCGGCGGGGUAGAGC-ACUGAU

Sac AGGGGCAAAAGACCAAUCUAGCCCGGUGAUAGCUAGUUCCCCCCGAAAUGCGUCCUAGCGCAGCCUCCCUAAAGGCAGCUCGCGGGGUAGAGUGACAGAU

Mka AGGGGUGAAAGGCCAAUCGAGGCCGGAGACAGCUGGUUCCCCCCGAAACGGCCCGCAGGUCGGCCCGGCCGGAGGUAGGUGGCGGGGUAGAGC-ACUGAU

Neq AGGGGCGAUAUACCAAUCGAGCCGGGUGAUAGCUGGUUCCCGCCGAAAUGGGCUGCAGUCCAGCCUCGGCGGAGGCACCCUCGGGUGUAGAGCGACGGAU

Iho AGGGGUGAAAGGCCAAUCUAGCUCGGUGAUAGCUGGUUCCCGCCGAAGUGGGUCUAAGCCCAGCGUCCCCGGAGGCGGGCCACGGGGUAGAGCUACUGAU

CON AGGGGcgAaAgaCCAAuCgAGcccGGuGAuAGCUgGUUCCCcCCGAAguGggucucAGccCaGCcuccccggAGgcagccggcgggGUAGAGc-ACuGAU

880 890 900 910 920 930 940 950 960

Eco UCGGCAA--GGGGGUCAUCCCGACUUACCA-ACCCGAUGCAAACUGCGAAUACCGGAG-AAUGUU—AUCACGGGAGACACACGGC-GGGUGCUAACGUCC

| | | | | | | | | 1200

S12

I310 K37 I1 K37 K108 K61I309

Pca CGGG-GGUGCGGGAGCCGAAA-GGCUCCGGCCCCCGGUCAAACUCCGAACCUGCCAGCGCCGUAGAAGGGGGGAGG-CGGGGGCG-GUGGGGUAAGCCUC

Mma AAGG-UGGUUAGGGGAAGAAA-UUCCUCGCUGUUUUGUCAAACUCCGAACUUGUCGUCGUCGC--AUGCUCCGAGU-GAGGGCAU-ACGGG-UAAGCUGU

Tte CGAG-GGCGCAGGGCCCGAAA-GGGUCCGGCCCUCGGUCAAACUCCGAACCCGCCGGAACCGUGGAAGGGGGGAGG-CGGGGCCA-GUGGGGUAAGCCUC

Sac CGGG-GGCUC--CAGGCGAAA-GCCUGGGGCUUCCGGUCUAACUCCGAACCCACGAGCGCCGAAGAAGGGGGGAGU-GGGUCACU-CGGCG-UAAGGUUG

Mka UCCCCGGUUA-GGGGCCGAGA-GGCCCCGCCGGGGAGUCAAACUCCGAACCCGCCACCGCCGUAGAAGGCCGGAGUUGGGGCCGG-CGG-G-UAA-GCCG

Neq UCCC-GUGGCAAGGGGGUUAA--CCCCACCACGGGAGUCCAACUCCGAAGCCCGAGGGGCCGUAGAUGCCGGGAGA-CGGGGGCG-GCGGA-UAAGCCGC

Iho GGGAGGUGCAGGGGGGCGUAGGCCCCCCGGCCUCCCGUCAAACUCCGAACCCGUGGCCGCCGUAGAAGGGGGCAGU-GGGGCGCCCCGGGG-UAAGCCCG

CON cggg-ggggcaggggccgaaa-gcccccggccuccgGUCaAACUCCGAAcccgccggcgcCGuaGAaGgggggAGu-ggGggccg-cgGgg-UAAGccuc

970 980 990 1000 1010 1020 1030 1040 1050

Eco GUCGUGAAGAGGGAAACAACCCAGACCGCCAGCUAAGGUCCCAAAGUCAUGGUUAAGUG-------GGAAACGAUGUGGGAAGGCCCAGACAGCCAGGAU
 | | | | | | | | | 1300

S12

I205K108 K61 K21/29 K87 I309 K30K29 K34K30K2

Pca CGCU-CCGAGACGGGAACAACCGAGACCGGGGUUAAGGCCCCCAAGUGCGGGCUUAGUGUCAA-UCUAAAAGGGCGUCCCCCGCCCAAGACAGCGGGGCC

Mma AUGU-CCGAGACGGGAAUAGCCGAGACUUGGGUUAAGGCCCCUAAGUGCCGAUUAAGUGUGA-ACAC-GAAGGGCGUCCUUGGUCUAAGACAGCAGGGAG

Tte UGGC-CCGAGACGGGAACAACCGGGACCGGGGUUAAGGCCCCUAAGUGCGGGCUAAGUGUCAA-CGGGUAAGGGCGUCCCCUGCCCAAGACAGCGGGGCC

Sac GGUG-GCAAAAGGGGAACAACCCAGACCUGGGUUAAGGCCCCAAAGUCCCGGCUAAGUGCCAA-CG--AAAAGGCGUCUCCAGCCUUAGACAGCGGGAAG

Mka CCGGUCUGAGAGGGGAAUAACCCAGACCGGGGUUAAGGCCCCAAAGUGCCGGCUAAGUGUUAA-AUAGAAAGGGAGUCCCCGGCCGAAGACAGCGGGGAG

Neq CGUU-CCGAGACGGGAACAACCGAGACCGGGGUUAAGGCCCCUAAGUGCCGGCUAAGUGGC----UG-GAAGGGUGUCCGCUCCCUAAGACACCGGGGAU

Iho GGGG-CCGAGAGGGGAACAACCCAGACCGGGGUUAAGGCCCCCAAGUGCCGGCUAAGUGCCAA-CCG-AAAGGGCGUCUCGGGCCUCAGACAGCGGGGAG

CON cggg-ccgAgAcGGGAAcAaCCgaGACcgGGGUUAAGGCCCCuAAGUgCcGgcUaAGUGucAA-ccg-aAAgGGcGUCcccggcCuaAGACAgCgGGgag

1060 1070 1080 1090 1100 1110 1120 1130 1140 1150

Eco GUUGGCUUAGAAGCAGCCAUCAUUUAAAGAAAGCGUAAUAGCUCACUGGUCGAGUCGGCCUGCGCGGAAGAUGUAACGGGGCU-AAACCAUGCACCGAAG
 | | | | | | | | | 1400

P42 T35 P42

K63 I152K15 K34 K15I312K63K89 K66 I152K89 K2 K87

Pca GUGGGCCUAACAGCAGCCAUCGGCUAAGCAACGCGUAACAGCGGACCCGCCGAGGCGGGGGGCCCCGAAGAUGUACAGGGACU-AAGCCCGCCGCCGAGA

Mma GUUGGCUUAGAAGCAGCCAUCCUUUAACGAGUGCGUAACAGCUCACCUGUCGAGAUCAAGGGCCCCGAAAAUG-GACGGGGCUAAA-UCGGCCGCCGAGA

Tte GUGGGCCUAACAGCAGCCAUCGGCCAAGCAACGCGUAACAGCGGACCCGCCGAGGCAGGGGGCCCCGUAGAUGUAGAGGGACUCAAGCCCGCCGCCGAGA

Sac GUGGGCCCAGCAGCAGCCAUCCUCUAAGGAGUGCGUAACAGCUCACCCGCCGAGGCUGGAGGCCCUAAAGAUUGGUCGGGGCUCAAGCCGGGCGCCGAGA

Mka GUAGGCUUAGAAGCAGCCAUCCUUUAAAGAGUGCGUAACAGCUCACCCGUCGAGGUCGGGGGCCCCGAAAAU-GGACGGGGCUGAAGCCGGCCGCCGAGA

Neq GUGGGACUAGUAGUGUCCAUCAUCUAAGGAGUGUGCAACAACUCACCCGGCGAGGGAGCGGGCCCCGCAGAUG-GACGGGCCU-AAGCCGGCCGCCGAGA

Iho GUGGGCCUAACAGCAGCCACCCUCUAAGGAGUGCGUAACAGCUCACCCGCCGAGGCCCGAGGCCCCGAAGAUUGGUCGGGGCUUAAGCCGGCCGCCGAGA

CON GUgGGccuAgcAGcagCCAuCcucuAAggAguGcGuAACAgCucACCcGcCGAGgccgggGGCCCcgaAgAUgggacGGGgCUcAAGcCgGcCGCCGAGA

1160 1170 1180 1190 1200 1210 1220 1230 1240 1250

Eco CUGCGGCAGCGACGC-----UUAU--GCGUUGU—UGGGUAGGGGAGCGUUCUGUAAGCCUGCGAAGGUGUGCUGUGAGGCAUGCUGGAGGUAUCAGAAGUG
 | | | | | | | | | 1500

T35* P20 T9 M5

K85 K84 K85

Pca CCCCGGCC-CGCGGGCC-GUU---GGCCCGCGUGGGGUAGGGGGGCGCGGCCGUG-GGGCAGAAGCCGGGCCGUGAGGUCCGGUGGACCCGCGGCCGACG

Mma CCCAAGGG-CACC-----GAAA-----GGUGAUCCUGUAGGGGGGCGUUCUGCGA-GGGCAGAAGUUCGGCUGUGAAGUCGAGUGGACCUCGUAGAAAUG

Tte CCCCGGGC-CUCCGGCC-GUU---GGCCGGAGUGCGGUAGGGGGGCGCGGCCGUG-GGGCAGAAGCCGGGCCGAGAGGUCCGGUGGACCCGCGGUCGACG

Sac CCCAGGAGGGGGU-CUCUACU---AGAGAUCCUCUGGUAGGGGGGCGCUGUGAUG-GGGUAGAAGGUGGGUCGUGAGAUCCACUGGACCCGUCACAGGUG

Mka CCCCGGGGCCGCGGGCC-GAU--GGCCCGCGGGCCGGUAGGGGGGCGCCCCGGCG-GCUCAGAAGCCGGGCCGUGAGGUCCGGUGGAGCCGUCGGGGACG

Neq CCCCGGGC-CGGGGAUCCGAU--GGAUCCCCGUGCGGUAGGCGGGCGUCCCGGUG-GCCUAGAAGGCGGGCCGUGAGGUCCGCUGGAGCCGCCGGGAAUG

Iho CCCCGGGC-CCCCCUCCCGGU--GGGAGGGGGUGCGGUAGGCGGGCGUCGGGGCG-GCCCAGAAGGCGGGCCGUGAGGUCCGCUGGAGCCGCUCCGAGUG

CON CCCcgGgc-cgccggcC-gau--GggccggggugcgGUAGGgGGGCGccgcggug-GgccAGAAGccgGGccGuGAggUCcggUGGAcCcgccgcggauG

1260 1270 1280 1290 1300 1310 1320 1330 1340

Eco CGAAUGCUGACAUAAGUAACG--AUAAAGCGGGUGAAAAGCCCGCUCGCCGGAA-GACCAAGGGUUCCUGUCCAACGUUAAUCGGGGCAGGGUGAGUCGAC
 | | | | | | | | | 1600

I25 I25 K58K102 K58

Pca AAGAUCCCGGCGGUAGUAGCAGCG-AAGAGGGGUGAGAAGCCCCUCCGCCGGAAAGGACCAGGGUUUCCUGGCAACUUCAAUAGGCCAGGAGUUAGCCGG

Mma AAGAUCCCGGUAGUAGUAACAGCAUAAGUGGGGUGAGAAUCCCCACCGCCGAA-GGGGCAAGGGUUCCACAGCAAUGUUUGUCAGCUGUGGGUAAGCCGG

Tte AAGAUCCCGGCGGUAGUAGCAGCG-AAGAGGGGUGAGAAGCCCCUCCGCCGGAAAGGACCAGGUUUUCCCGGCAACUACAAUAGGCUGGGAGUUAGCCGG

Sac CAGAUCCCGGCGGUAGUAACAGCG-AAGGGGGGUGAGAAUCCCCCUCGCCGGA-AGGGCAAGGGUUUCCCGGCAACGUUCGUCAGCCGGGAGUUAGCCGG

Mka AGAAUCCCGCCGGUAGUAGCAGCA-AAGCGGGGUGAGAAUCCCCGCCGCCGGA-GGGGCCAGGGUUCCUCGGCAAUGUUCGUCAGCCGAGGGUAAGUCGG

Neq AGAAUCCCCGCGUGAGUAGGAGCA-AAGAGGGGUGAGAAGCCCCUCCGCCGAAAGGGAAAAGGGUUCCCUGGCAAUGACCAUCAGCCAGGGGUGAGUCGG

Iho CCGAUCCCGGCGGUAGUAACAGCG-AAGAGGGGUGAGAAUCCCCUCCGCCGGAAAGGGCAAGGGUUCCCCGGCAACUGUCAUAGGCCGGGGGUGAGUCGG

CON aagAUCCCggcgguAGUAgcAGCg-AAGaGGGGUGAGAAuCCCCucCGCCGgAAaGGgcaAGGgUUcCccgGCAAcguucaUcaGCcggGgGUuAGcCGG

1350 1360 1370 1380 1390 1400 1410 1420

Eco CCCUAAGGCGAGGCC-GAAAGGC-GUAGUCGAU-GGGAAACAGGUUAAUAUUCCUGUACUUGGUGU-UACU--------------------GCGAAGGGG
 | | | | | | | | | 1700

P42 P133 P105 P133* P105

K102 K43 K43 K22 K24

Pca UCCUAAGGCGGGGCCUAAUAGGCACCCGCCGAAAGGGAAACGGGUUAAUAUUCCCGUGCCGCGGGGGUAGGU-UCUGCGGCAACGC-AGG-CCCCGUCCC

Mma UCCUAACUCUCGAGUUAACUUCUUUGAGAGGAAAGGGAAACAGGUUAAUAUUCCUGUGCCAUCUAAAUACGC----GUGGCAACAC-UAG-GUUAGUUUC

Tte UCCUAAGGCGGGGCCUAGCUGGCACCCGCCGAAAGGGAAACGGGUUAAUAUUCCCGUGCCGCGGGGGUAGGU-UCUGCGGCAACGC-AGG-CCCCGUCCC

Sac UCCUAAGGUAGGGCCUAAUAGGUACCUACCGAAAGGGAAAGGGGUUAACAUUCCCCUGCCUCCCGGGUAGGU----GCGGUAACGC-AAG-CCAGACUCC

Mka CCCUAAGGCCGGCGGUAACACCGACCGGCCGAAAGGGAAACGGGUUAAUAUUCCCGUACCGCGGGGGUACGC----CCGGCAACGGGAAG-CCCGGCGGC

Neq UCCUAAGGCCGGCGGUAACUCCGCUCGGCCGAAAGGGAAAGGGGUUAAUAUUCCCCUACCGCCCGGGUAGGC----GCGGCAACGCCAAG-CCCUACUCC

Iho UCCUAACCCGCCUCCCAACUGGAGCGCGGGGAAAGGGAAGCCGGUUAAUAUUCCGGCACCGCGGGGGUAGGU----GCGGCAACGC-AAG-CCCCACCUC

CON uCCUAAggcggggccuAacuggcacccgccGAAAGGGAAacgGGUUAAuAUUCCcgugCCgcgggggUAgGu----gcGGcAACgc-aaG-ccccgcccC

1430 1440 1450 1460 1470 1490 1500 1510 1520

Eco GGACGGAGAAGGCUAUGU-UGGCCGGGCGA--CGGUUGU----CCCGGUUUAAGCG...UUUCCAGGCAAAUCCGGAAAAUC-----AAGGCUGAGGCGU

| | | | | | | | | 1800

K55/92 K92 I309 K54

Pca CGACGCCUCGGGAUAGGG-CGGGCGGGACUGCCGUCCCGCUUAACC-GUCGAAGGCCGGGGAGUGCCGUAAUGGCGAGAACCGGCCGAAGGCGGGAAUAG

Mma CGACGCUUCUGGGUAGGC-UGAGUGUUCUUGUCUGGGCAUUCAAGC-UUAUAAGUCCGGGGAGAGUUGUAAUAACGAGAACCGGAUGAAAGAGUGAUGAG

Tte CGACGCCUCGGGAUAGGG-CGGGCGGGACCACCGUCCCGUUUAACC-GCUGAAGGCCGGGGAGUGCCGUAAUGGCGAGAACCGGCCGAAGGCGGGAAUAG

Sac UGACGGAUUGGGGUAGGG-AGAGUAGGACCACCGUCCUACCCAAGC-ACUCAAGCCCUUGGAGAGCCGUAACGGUGAGAAGAGGGCGAAGGUGUGAUGGG

Mka CGACGCCUCGGGGUAGGCCGGGCGGGGCUGUC-GCCCCGUCCAACC-GGUGAAGGCCGGGGAGUCCCGUAAUGGGGAGAACCGGCCGAAGCCGGGAAGGG

Neq CGACGCCUGGGGCUAGGC-CGACCGCGGAUUUCCCGCGGCCAACCGGGAUAGGGCCGGGGAGUGCCAUUAUGGCGAGAACCGGCCUAAACCCGGGACGGG

Iho CGACGCCUCGGGGUAGGC-GGACCGGGGCUGUCGCCCCGGCUAACCGUCCGAAGGCCCUGGAGGGCCGUAACGGCGAGAAGGGGCCGAAGGCGGGAAGGG

CON cGACGccUcgGGgUAGGc-cGagcgggacugcCgccccgcccAacc-gcugaaGgCcggGgagugccgUAauggcgagAaccGgccgAAggcGgGAaggG

1550 1560 1570 1590 1600 1610 1620

Eco GAUGACGA......GAAGCAACAAAUGCCCUGCUUCC-AGGAAAAGCCU.....AUCAGGUAACAUCAAAUCGUACCCCAAACCGACACAGGUGGUC-AG
 | | | | | | | | | 1900

K109 I31 K109 K103 I64 I31 K109

Pca CCGGGGGU-UUCCCCGGUCCGCCCGACUCCUGGGGCCCGUGAAAAGGGGACGGGGAACGAGCCCCCGCGCCCGUACCGAGAACCGACGCAGGUGCUCCUG

Mma CUCUCCGU-UAGGAGGGUUCGGCCGAUCUCUGGAGCCCGUGAAAAGGGAAUUAACAAGGAUUUUAGAUGUCCGUACCCAGAACCGACACUGGUGCCCCUA

Tte CCGGGGGU-UUCCUCGGUCCGCCCGACUCCUGGGGCCCAUGAAAAGGGGACGGGGAACGAGCCCUCGCGUCCGUACCGAGAACCGACGCAGGUGUUCCUG

Sac CCUUCCGU-UAGGAGGGUUCUCCUGAUCCCUAGUCCCCAUGAAAAGGGAGUCUGGAACGAUCCCGGGAGACCGUACCUAGAACCGACACUGGUGCCCCUG

Mka CCGCCCGUUAUGGGCGGUCCGGCCGAUCCCCGGGGCCCGUGAAAAGGCCGCCGGGAAGGAUCCCCCGCGACCGUACCGAGAACCGACACAGGUGCCCCUG

Neq GCCCCCGU-AUGGGGGCUUCGGCCGAGGCCCUGGGCCCAUGAAAAGGGAGUAGGGAACGAUCCCGGGCGACCGUACCGAGAACCGUGACAGGUU-CCCUG

Iho GCCCCCGU-AUGGGGGCUUCCGCCGAUCCCUGGGGCCCAUGAAAAGGGGGUGGGGAACGAUCCCCCGCGCCCGUACCGAGAACCGACGCAGGUGCCCCUG

CON ccccccGU-uuggggGgUuCggCcGAuccCugGggCCCaUGAAAAGGgaguggggAAcGAucccccgcGaCCGUACCgAGAACCGacaCaGGUgccCCUg

1630 1640 1650 1660 1670 1680 1690 1700 1710 1720

Eco GUAGAGAAUACCAAGGCGCU-UGAGAG-AACUCGGGUGAAGGAACUAGGCAAAAUGGUGCCGUAACUUCGGGAGAAGGCACGCUGAUAUGUAGGUGAAGC
 | | | | | | | | | 2000

M5 P38N21T51

I114 I169 I114 I169

Pca GGUGAGAAGCCCAAGGCGGCUCGGGU-GACCCCGGGCCAGGGAACUCGGCAAAUUGGCCCCGUAACUUCGGGAGAAGGGGUGCCUGCGGUCUUGGGG---

Mma GGUGAGUAUCCUAAGGCGUAGCGGAU-GAAUCUAGUCGAGGGAAGUCGGCAAAUUGGCUCCGUAACUUCGGGAGAAGGAGUGCCAGUGAUCUUGUU----

Tte GGUGAAAAGCCCAAGGCGGCUUGGGUUUACCCCAGGCCAGGGAACUCGGCAAAUUGGCCCUGUAACUUCGGGAGAAGGGGUGCCUGCGGUCUUGGGG---

Sac GGUGAGAAGCCCAAGGCGUCUGAGGGGUAACCCAGGCUAGGGAACUCGGCAAAUUAGCCCCGUAACUUCGGGAGAAGGGGUGCCUAUCGUGGUUU-----

Mka GGUGAGAAGCCUAAGGCGCGGCGGGG-UAACCCGGCCGAGGGAAAUCGGCAAAUUGGCCCCGUAACUUCGGGAGAAGGGGUGCCCGCGGCCCUGACCCCG

Neq GGUGAGCAGCCUAAGGCGGUGGGGUA-UAACCUGGCCGAGGGAAAUCGGCAAAUUGGCCCCGUAUCUUCGGUAGAAGGGGUGCCUGCGGUCGUGGGG---

Iho GGUGAGAAGCCCAAGGCGGCUGGGGGUUAACCCGGGCCAGGGAACUCGGCAAAUUGGCCCCGUAACUUCGGGAGAAGGGGUGCCUGCGGUCCUGGGG---

CON GGUGAgaAgCCcAAGGCGgcucgGgg-uAacCcgGgCcAGGGAAcUCGGCAAAUUgGCcCcGUAaCUUCGGgAGAAGGgGUGCCugcggucuUgggg---

1730 1740 1750 1760 1770 1780 1790 1800 1810

Eco GACUUGCUCG------UGGAGCUGAAAUCAGUCGAAGAUACCAGCUGGCUGCAACUGUUUAUUAAAAACACAGCACUGUGCAAACACGAAAGUGGACGUA
 | | | | | | | | | 2100

S2 N16 P21S4

I76K36 K76 I96K36 K20

Pca ----UAUAC-------CCCCGGGACCGCAGGUCGCAGUGGCAAGGGGGACCUGACUGUUUAACAAAAACAUAGGUCCCCGCGAGCCCGUAAGGGUGUGUA

Mma ----UAUA---------UAUGGGAUCGCUGGUCGCAGUGACCAGGGAGGUCCGACUGUUUAAUACAAACAUAGGUCUUAGCGAGCCUGAAAAGGUGUGUA

Tte ----UUCAC-------CCCUGGGACCGCAGGUCGCAGUGACAAGGGGGACCUGACUGUUUAACAAAAACAUAGGUCCCCGCGAGCCCGUAAGGGUUUGUA

Sac ----AACA----------AAGCCACGAUAGGUCGCAGUGACCAGAGGGACCUGACUGUUUAAUAAAAACAUAGGUCCCCGCUAGCCCGAAAGGGUGUGUA

Mka AGGGUCGA-ACCUCGGGGGAGGGGCCGCGGGUCGCAGUGACUAGGGGGGGCCGACUGUUUAAUAAAAACAUAGGUCCCGGCUAGCCCGAAAGGGCUGGUA

Neq -----UAA--------UCCUGCGACCGCAGGUCGCAGUGACAAGGGCGGGCCGACUGUUUACUAAAAACAUAGCCUCCGGCAAGCCCGAAAGGGUGUGUA

Iho ----CCCAC-------CCCUGGGACCGCAGGUUGCAGUGCCUAGGGGGGCCUGACUGUUUAAUAAAAACAUAGGUCCCCGCAAGCCCGAAAGGGUGUGUA

CON ----uacA--------cccuGggaccgcaGGUcGCAGUGaCaAGgGgGgcCuGACUGUUUAauAaAAACAUAGguccccGCgAGCCcGaAAgGGuguGUA

1820 1830 1840 1850 1870 1890 1900 1910 1920 1930

Eco UACGGUGUGACGCCUGCCCGGUGCCGGAAGGUUAAUUGAU....CAAGCG..CGAAGCCCCGGUAAACGGCGGCCGUAACUAUAACGGUCCUAAGGUAGC
 | | | | | | | | | 2200

P13S4 P21 T33N26 P33N26 S125T40M7N11S113N22S20M4

P124T33 P124 P33 N16 P103-109 P112

K86 K20K48 K22 K22 K86 K111 K77/95 K47 K69K43K48 K47 K43

I103 I208 I415 I205I314/96 I208 I128I109I441I415*I91 I201 I418

Pca CGGGGGCUGAAUCCUGGCCACUGGCGGUACGUGAACCCCGGGUACAACCGGGCGAAGCGCCGCUGAAGGCCGGGGGUAACUCUGACCCUCUUAAGGUAGC

Mma CUAAGGCCGACGCCUGCCCAGUGCUGGUACGUGAACCCCGGUUCCAACCGGGCGAAGCGCCAGUAAACGGCGGGGGUAACUAUAACCCUCUUAAGGUAGC

Tte CGGGGGCUGAAUCCUGGCCACUGGCGGUACGUGAACCCCGGGUCCAACCGGGCGAAGCGCCGCUGAAGGCCGGGGGUAACUCUGACCCUCUUAAGGUAGC

Sac CGGGGGCUAAAUCCUGGCCACUGGUGGUUGGUUAAAUCCGGGUUCAACCGGGCGAAGCCCCACCGAAGGCCGGGGGUAACUCUGACCCUCUUAAGGUAGC

Mka CCGGGGCCGACGCCUGCCCAGUGCCGGUACGUGAAGCCCGGGUACAACCGGGUGAAGCGCCGGUAAACGGCGGGGGUAACUAUAACCCUCUUAAGGUAGC

Neq CCGGACGUGACGCCUGCCCAGUGCCGGUACCUGAACCCCCCGUCCAAGGGGGCUAAGGGCCGGUAAACGGCGGGAGUAACUGUAACUCUCUUAAGGUAGC

Iho CGGGGGCCGAAUCCUGGCCACUGGCGGUCCGUGAAACCGGGGUUCAACCCGGCGAAGCGCCGCUGAAGGCCGGGAGUAACUCUGACUCUCUUAAGGUAGC

CON CgggggcugAauCCUGgCCAcUGgcGGUacgUgAAccCcgggUcCAAccgGGcgAAGcgCCgcugAAgGcCGGGgGUAACUcUgACcCUCUUAAGGUAGC

1940 1950 1960 1970 1980 1990 2000 2010 2020 2030

Eco GAAAUUCCUUGUCGGGUAAGUUCCGACCUGCACGAAUGGCGUAAUGAUGGCCAGGCUGUCUCCACCCGAGACUCAGUGAAAUUGAACUCGCUGUGAAGAU
 | | | | | | | | | 2300

S125P40 T30T40 S20P40 T30S106 T51 P44 M5N2 P44T43

P54 P112 P54

I109 I91 I441 I418 K69 I308 K111 I308 I201*I114I404K112 I114 I423

Pca CAAAUGCCUUGCCGGGUAAGUUCCGGCGUGCAUGAAUGGAUCAACGAGGUCCCCACUGUCCCGGCCCGGGGCCCGGCGAACCCACC-UCCAGGUGCACAG

Mma GAAAUUCCUUGUCGGGCAAGUUCCGACCUGCAUGAAUGGCGUAACGAGACCUCCACUGUCCCCGACUAGAAUCCGGUGAACCUACCAUUCCGGCGCAAAG

Tte CAAAUGCCUUGCCGGGUAAGUUCCGGCGUGCAUGAAUGGAUCAACGAGGUCCCCACUGUCCCGGCCUGGGGCCCAGCGAACCCACC-UCCAGGUGCACAG

Sac CAAAUGCCUUGCCGGGUAAGUUCCGGCGCGCAUGAAUGGAUCAACGCGGUCCCUACUGUCCCAGCCUGGGGCCUCGUGAACGCCCUGAGCCGGUGCACAG

Mka GAAAUGCCUUGCCGGUUAAGUACCGGCUUGCAUGAAUGGCGUAACGAGCCCCCCACUGUCCCCGGCCGGGACCCGGUGAACCCGCCAUCCCCGUGCAGAG

Neq GUAGUCCCUUGCCGGUUAAAUGCCGGCUC-UGCGAACGGCGUAACGAGCUCGCCACUGUCCUCGGCCAGGGCCCCCUGAAAACGCCGUUCCCGUGGAUAG

Iho CAAAUGCCUUGCCGGGUAAGUUCCGGCGCGCAUGAAUGGAUCAACGAGGUCCCCACUGUCCCGGCCCGGGGCCCCGUGAACCCCCUGAGCCGGUGCACAG

CON caAaUgCCUUGcCGGguAAgUuCCGgCguGcauGAAuGGaucAACGaGguCcCcACUGUCCccGcCcgGggcCccguGAAcccaCcgucCcgGuGcAcAG

2040 2050 2060 2070 2080 2090 2100 2110 2120 2130

Eco GCAGUGUACCCGCGGCAAGACGGAAAGACCCCGUGAACCUUUACUAUAGCUUGACACUGAACAUUGAGCCUUGAUGUGUAGGAUAGGUGGGAGGCUUU--
 | | | | | | | | | 2400

P58T11T43 S107 T11N2P58

I404 I27

Pca UCCUGGGACCCCCGACGGGGCGAGAAGUCCCUAUGGAGCUUCACAGCAGCCUGUCGUUGCGGGGGGGCGGGGGGUGCAGAGCGUAGGUGGGAGCGAU---

Mma GCCGGAGACUUCCAGUGGGAAGCGAAGACCCCGUGGAGCUUUACUGCAGCCUGUCGUUGGGGCAUGGUUGUGAGUGUACAGUGUAGGUGGGAGCCAU-C-

Tte UCCUGGGACCCCCGACGGGGCGAGAAGUCCCUGUGGAGCUUCACUGCAGCCUGUCGUUGCGGAGGGGCGGGGGGUGCAGAGCGUAGGCAGGAGCAAU---

Sac UCCGGCAUCUCCCUACACCGAGAGAAGACCCCGUGGAGCUUCACCGCAGCCUGGCGUUGUCCCUCGGGCGUUUAUGCGUAGAGUAGGUGGGAGGGGU-C-

Mka GCGGGGGACCCCCGGCGGGAAGCGAAGACCCCGUGGAGCUUUACCGCAGCCUGCCGUUGGGGCACGGCUGCGGGUGUAUAGCGUAGGUGGGAGCCGU-UA

Neq GCGGGAGAGGCCCGGUGGGAAGAGAAGACCCCGUGAAGCUUAACCGCAGCCUGUCGUUGCCCCGUUGUCCCCCACGCUCAGCGUAGCGGGGAGCGAU---

Iho UCCGGCAACCCCCCGUAGGGAGAGAAGACCCCGUGGAGCUUUACUGCAGCCCGGCGUUGCCCCCCGGGUGGGGGUGCGUAGCGUAGGUGGGAGCC-GGU-

CON uCcgGggacccCCggcggggaGaGAAGaCCCcgUGgAGCUUcACcGCAGCCuGuCGUUGcggcgcgGcuggggguGcauAGcGUAGgugGGAGccau-c-

2140 2150 2160 2170 2180 2190 2200 2210 2220

Eco GAA--GUGUGGACGCCAGUCUG-CAUGGAGCCGACCUUGAAAUACCACCCUUUAAUGUUUGAUGUUCUAACGUUGA--CCCG---UAAUCCGGGUUGCGG
 | | | | | | | | | 2500

N12

K59 I31 K78 K40 K13

Pca GAA--ACGGGGUCUCCGGGCCC-CGUGGAUGCGACCCUGGAACACCACCCACUCUCCGCCCCUCCGCUUACCCGCC--GCAA----GGCGGGGACAGCGG

Mma GAA--ACCUUUUCGCCAGGAAA-GGUGGAGGCGACCCUGGGACACCACCCUCUCAUGACCAUGUUCCUUACCCU-----UUU------AGGGGACACCGG

Tte GAA--ACGGGGUCUCCGGGCCC-CGUGGAUGCGGUCCUGGAACACUGCCCACUCUCCGCCCCUCCGCUAACCCGGG--GCAA----CCCGGGGACAGCGG

Sac GAA--CCUGUCCUUUCGGGGGC-AGGGGACCCGAAAGUGAAACACCACCCAUGGACGCUCGAGGGACUAACCUCUC--GAAA----GAGAGGAACAACGU

Mka CUCAGGGGCGGCCGCCAGGCCGUCCCCGAGGCGCCCAUGGAACACCACCCUCCCGCGGCUGUGCCCCUAACCCGGGCCGAGAA-GGCCCGGGGACAGCGG

Neq GAA--CCGGCCCCUCCGGGGGU-CGGGGAUGCGCCCAUGAGACACCCGCCGUGGGGGACAACGGGGCUAACCCCG----AAA------GGGGGACAGCGG

Iho GAA--CCGGCCCCUCCGGGGGU-CGGGGAGGCGCCCAUGAAACACCACCCACCCUCACCCGGGGGGCUUACCCCG---GGGGAAGCCCGGGGGACAGCGC

CON gaa--acgggcccucCgGGccc-cgggGAggCGaccaUGgaACACcacCCacucucggccgcgccgCUaACCccgc--Gaaa----cccgGGgACAgCGg

2240 2250 2260 2270 2280 2290 2300 2310 2320 2330

Eco AC...GGGUAGUUUGACUGGGGCGGUCUCCUCCUAAAGAGUAACGGAGGAGCACGAAGGUUGGCUAAUCCUGGUCGGACAUCAGGAGGUUAGUGCAAU-G

| | | | | | | | | 2600

P128 P36T7P128T22 T7P36 P109N12 P109T26*

K40 K13/45 I401K85 K85 I160K87 K45 K33K61I86

Pca CAGGCGGGCUGUUCGGCUGGGGCGGCACACCCCUGAAAAGAUAUCGGGGGUGCCCAAAGCUCGGCUCAGGCGGGUCAGAAAUCCGCCGUAGAGUGUAAGG

Mma UAGGUGGGCAGUUUGGCUGGGGCGGUACCCUCCUAAAAAUGCAUCAGGAGGGCCCAAAGGUUGGCUCAAGCGGGUCAGGACUCCGCUGUUGAGUGUAAGG

Tte CAGGCGGGCAGUUCGGCUGGGGCGGCACACCCCUGAAAAGAUAUCGGGGGUGCCCAAAGCUCGGCUCAGGCGGGUCAGAACUCCGCCGUAGAGUGUAAGG

Sac CAGGUGGGCGGUUCGGCUGGGGCGGCACUCCCGCGAAAAGAUAACACGGGAGCCCAAAGGUCGGCUCAGGCGGUACAGAACGCCGCCGUAGAGCGCAAGG

Mka UUGGUGGGCGGUUUGGCUGGGGCGGCACGCCCCCGAGAAGGCAUCGGGGGCGCCCAAAGGUCGGCUCAGGCGGGUCAGAAAUCCGCCGUAGAGUGCAAGG

Neq CAGGUGGGCGGUUUGGCUGGGGCGGCACACCGCCGAAAAGAUAUCGGCGGUGCCCAAAGGUCGGCUCAGGCGGGUCAGAAAUCCGCCGUAGAGUGCAAGG

Iho CGGGUGGGCAGUUUGGCUGGGGCGGCACGCCCGCGAGAGGGUAACACGGGCGCCCUAAGGUCGGCUCAGGCGGGUCAGAAACCCGCCGUAGAGUGCAAGG

CON caGGuGGGCaGUUuGGCUGGGGCGGcACaCccccgAaAagauAuCggggGuGCCCaAAGgUcGGCUCAgGCGGguCAGaAauCCGCcGUaGAGuGcAAGG

2340 2350 2360 2370 2380 2390 2400 2410 2420

Eco GCAUAAGCCAGCUUGACUGCGAGCGUGAC-----GGCGCGAGCAGGUGCGAAAGCAGGUCAUAGUGAUCCGGUGGUUCUG—AAUGGAAGGGCCAUCG..
 | | | | | | | | | 2700

T26 S128M3P16

K87K84 K53 I127K33 K51 K84 I127 K51 K57 I144 I86 I52 I473 I425K126

Pca GCAAAAGCCGGGCUGACUGGGCCCUUGAAC--GCAAGGGGCCCAGGCGGGAAACCGGGGCCUAGAGAACGCUCGUGCCCCCACCAGUGGGGGCCGGGCAU

Mma GCAAAAGCCAGCCUGACUUUGUUGCCAACAAA-A-CGCAACGAAGAGGCGAAAGCCGGGCCUAACGAACCCCUGUGCCUCA-CUGAUGGGGGCCAGGGAU

Tte GCAAAAGCCGGGCUGACUGCGCCCUUGAAC--GCAAGGGGCGCAGGCGGGAAACCGGGGCCUAGAGAACGCUCGUGCCCCCACCAGUGGGGGCCGGGCAU

Sac GCAAAAGCCGGCCUGACGUGACCCUUCCAA--GUACGGGGUCACGACGCGAAAGCGGGGCCUAGCGAACGCUCGUGCCCCCACACGUGGGGGCCGGGCAU

Mka GCAAAAGCCGGCCUGACUGGGUCCCGCACA--AUAAGGGACCCAGGCGGGAAACCGUGGCCUAGCGAACCCCGGUGCCGCC-CCGGUGGCGGCCCGGGAU

Neq GCAAAAGCCGGCCUGACACGGCCCUUUA-----CAAGGGGCCGUGGCGGGAAACCGCGGCCUAGCGAACCCCGGUAGCCCC-CGUGUGGGGCUUCGGGAC

Iho GCAAAAGCCGGCCUGACCGGACCCCUAAAG--GCAGGGGGUCCGGCCGGGAAACCGCGGCCUAGCGAACGCUCGUGUCCCCCCUGGUGGGGGCCGGGCAU

CON GCAAAAGCCgGcCUGACugggcccuuaaaa--gcAaGgggcccaGgcGgGAAAcCggGGCCUAgcGAACgCucGUgcCcCcaCcggUGGgGgccgGGcAu

2430 2440 2450 2460 2470 2480 2490 2500 2510 2520

Eco CGGAUAAAAGGUACUCCGGGGAUAACAGGCUGAUACCGCCCAAGAGUUCAUAUCGACGGCGGUGUUUGGCACCUCGAUGUCGGCUCAUCACAUCCUGGGG
 | | | | | | | | | 2800

N14S128 M1 T17 S17 S11T50 P17N3 T17 T50N4S7 P110

K16 K7 K126K17 K38K28 K56/103 K69 K16 K45K7

I425 I414 I132 I204 I165 I132 I414 I204 I165I473

Pca GACAGAAAAGUUACCCUAGGAAUAACCGGCUCGUCGCGGGUGAGAGUCCCCAUCGACCCCGCGGUUUGGUACCCAGACGUCGUCUCUUCCCAUCCUGGCG

Mma GACAAAAAAGCUACCCCGGGGAUAACAGAGUUGUCGCGGGCAAGAGCCCAUAUCGACCCCGCGGCUUGCUACCUCGAUGUCGGUUUUUCCCAUCCUGGGU

Tte GACAGAAAAGUUACCCCAGGAAUAACCGGCUCGUCGCGGGUGAGAGUCCCCAUCGACCCCGCGGUUUGGUACCCAGACGUCGUCUCUUCCCAUCCUGGCG

Sac GACAGAAAAGUUACCCCGGGGAUAACAGGGUCGUCGCGGGCGAGAGCUCACAUCGACCCCGCGGUUUGCUACAUCGAUGUCGGCUCUUCCCACCCUGGAG

Mka GACAGAAAAGCUACCCCGGGGAUAACAGGGUGGUCGCGGGCAAGAGCCCACAUCGACCCCGCGGCUUGCUACCUCGAUGUCGGCUCUCCCCAUCCUGGCG

Neq GACGGAAAAGCUACUCCGGGGGUAACAGCUUGGUCGCGGGCGAAAGUUCCCAUCGACCCCGCGGUUUGAGACCUCGAUGUCGGCUCUUCUCACCCUGGCC

Iho GACAGAAAAGUUACCCCGGGGAUAACAGAGUCGUCGCGGGCGAGAGCCCACAUCGACCCCGCGGUUUGCUACCUCGAUGUCGGCUUUUCCCAUCCUGGGG

CON GACagAAAAGuUACcCcgGGgaUAACaGggUcGUCGCGGGcgAgAGccCacAUCGACCCCGCGGuUUGcuACcucGAuGUCGgcUcUuCcCAuCCUGGcg

2530 2540 2550 2560 2570 2580 2590 2600 2610 2620

Eco CUGAAGUAGGUCCCAAGGGUGAUGCUGUUCGCCAUUUAAAGUGGUACGCGAGCUGGGUUUAGAACGUCGUGAGACAGUUCGGUCCCUAUCUGCCGUGGGC
 | | | | | | | | | 2900

T5 N10S7 T45 S105 S13* T45 T52S105*T17 S3 T52N10S13T5T50

P110 P115 P37 P115 P37 P30 P17 P30

K64I6 I160K38 I33K17 K64 I124K103 K56 I124/I27 K68 I411 I6K3 K80

Pca GUGCAGCAGCCGCCAAGGGUGGGGCUGCCCGCCCAUUAAAGGGGAACGUGAGAUGGGUUCAGACCGUCGCGAGACAGGUCGGUCUCUACCUGUCGGGGGC

Mma CUGCAGCAGGACCCAAGGGUGGGGCUGUUCGCCCAUUAAAGGGGAUCAUGAGCUGGGUUUAGACCGUCGUGAGACAGGUUGGUUGCUAUCUGCUGGAUGU

Tte GUGCAGCAGCCGCCAAGGGUGGGGCUGCCCGCCCAUUAAAGGGGAACGUGAGAUGGGUUCAGACCGUCGCGAGACAGGUCGGUCUCUACCUGUCGGGGGC

Sac GUGCAGCUGCCUCCAAGGGUAGGGCUGCCCGCCCGUUAAAGGGGAGCGUGAGCUGGGUUUAGACCGUCGCGAGACAGGUCGGACUCUAAGGGUAGGGAGU

Mka GUGCAGCAGCCGCCAAGGGUGGGGUUGUUCGCCCAUUAAAGGGGAACGUGAGCUGGGUUUAGACCGUCGUGAGACAGGUCGGUCGCUAUCUGCCGGGGGU

Neq GUGCAGCAGCGGCCAAGGGUGGGGGUGUUCACCCAUUAAAGAGGAACGUGAGCUGGGUUUAGUACGCCGCGAGGCAGUGUGGAUUCUAUCUACCGGGCCU

Iho GUGCAGCAGCCCCCAAGGGUGGGGCUGCCCGCCCAUUAAAGGGGAACGUGAGCUGGGUUUAGACCGUCGUGAGACAGGUCGGACUCUACCCACGGGGGGU

CON gUGCAGCaGccgCCAAGGGUgGGGcUGccCgCCCaUUAAAGgGGAaCgUGAGcUGGGUUuAGacCGuCGcGAGaCAGgucGGucuCUAccugccGGgggu

2630 2640 2650 2660 2670 2680 2690 2700 2710 2720

Eco GCUGGAGAACUGAGGGGGGCUGCUCCU-AGUACGAGAGGACCGGAGUGGACGCAUCACUGGUGUUCGGG...AUGCCAAUGGCAC-UGCCCGGUAGCU..
 | | | | | | | | | 3000

P115 T17

K3 K32* K80 K66 K8I1 I303

Pca GCUGGCCGCCUGAGGGGAAGGUGCCCUCAGUACGAGAGGAACGGGGCGCCGCGGCCUCUAGUGUACCGGUUGUCCGGCAGGGCAC-UGCCGGGCAGCCAC

Mma GUUGGCUGUCUGAGGGAAAGGUGGCUCUAGUACGAGAGGAACGGGCCGUCGGCGCCUCUAGUCGAUCGGUUGUCUGACAAGGCAU-UGCCGAGCAGCCAC

Tte GUUGGCCGCCUGAGGGGAAGGUGCCCUCAGUACGAGAGGAACGGGGCGCCGCGGCCUCUAGUCUACCGGUUGUCCGGCAGGGCAC-UGCCGGGCAGCCAC

Sac GCGGACCGCUUGAGGGGAAGGAACCCCUAGUACGAGAGGAACAGGGUUCCGGGGCCUCCAGUUUACCGGUUGUCCGGUAGGGCAA-UGCCGGGCAGCCGC

Mka GUUGGCCGCCUGAGGGGAAGGACCGCCUAGUACGAGAGGAACGGCGGUCCGCGGCCUCUGGUGGACCGGCUGUCCGGCAGGGCAA--GCCGGGUAGCUAC

Neq GUUCGGCGGCUGAGGGGAAGGACGGCCUAGUACGAAAGGAACGGCCGUCCGGCGCCUCUAGUCGACGGGGUGUCCGUAUGGGCAG-C-CCCGGCAGCCAC

Iho GUCGGCCGCCUGAGGGGAAGGUGCCCUCAGUACGAGAGGAACGGGGCGCCCCGGCCUCUGGUGUACCGGUUGUCCGGCAGGGCAGA-GCCGGGCAGCUAC

CON GuuggccGccUGAGGGgAAGGugccccuAGUACGAgAGGAACgGggcgcCgcgGCCUCuaGUcuAccGGuUGUCcGgcagGGCAa-uGCCggGcAGCcaC

2730 2740 2750 2760 2770 2780 2790 2800 2810

Eco UGCGGAAGAGAUAAGUGCUGAAAGCAUCUAAGCACGAAACUUGCCCCGAGAUGAGUUCUCCCUGACC-----CUUUAAG-----GGUCCUGAAGGAACGU
 | | | | | | | | | 3100

T40P122S22 P122S22 P107 S22

I25K8 I422 K32 K115 K66I422K66 I311K115 I303

Pca GCCGUGGGGGAUAACCGCUGAAAGCAUCUAAGCGGGAAGCCCUCCCCGAGACGAGGCGGCCGUUGCCCUGGGGGCAACCCCGGGGCACGAGGGCUCCCGU

Mma GCGCCAAGAGAUAAGAGCUGAAAGCAUCUAAGCUCGAAAUUCAUCCUGAAAAUAGACAGCCGUUUCC------UUCG------GGAACGAGAACAUCUGU

Tte GCCGUAGGGGAUAACCGCUGAAAGCAUCUAAGCGGGAAGCUCCCCCCGAGACGAGGCGGCCGUUGCCCUGGGGGGCAACCCCUGGGGCACGAGGGCUCCC

Sac GCCCUGAGGGGUAACCGCUGAAAGCAUCUAAGCGGGAACCCCUCCCCUAAAAGAGGCGGUCA-GGC-------GUUA-------GCCGGGGCCUUCCCCU

Mka GCCGUAUCCGAUAAGGGCUGAAAGCAUCCAGGCCCGAAGCGGUCCCCGAAAAUAGGCGGCCGU------------------------UGAGGCCUCGGGU

Neq GCGCUAGGGGGUAAGGGCUGAAUGCAUCUAAGCCCGAACCCCUCCCCGAAAAGAGCCG-CCGAU-------------------------CCCCCUCGGGU

Iho GCCGGAAGGGGUAACCGCUGAAAGCAUCUAAGCGGGAACCCCACCCCGAAAAGAGGCGGCCAUU------------------------GAGGGCUCCCGU

CON GCcguaaggGaUAAccGCUGAAaGCAUCuAaGCggGAAccccucCCcgAaAagAGgCgGcCguugC-------guca-------Gcacgaggccucccgu

2820 2840 2850 2860 2870 2880 2890 2900

Eco UGAAGACG..GUUGAUAGGCCGGGUGUGUAAGCGCAGCGA-UGCGUU----------------GAGCUAACCGGUACUAAUGAACCGUGAGGCUUAACCUU

| | | | | | | | | 3200

P50 P50

K31 K88 K21 K122 K88K31 K31

Pca AGAAGACGGGGUUGAUGGGGGGGCGGUGUAACCCCCGAGGG---UUU-----CCCGAGGGGA-GAGCCGGCCCCUCCCAAUCGCCCGAGCGUGCGGGGCG

Mma AGAAGACAGGUUUGAUAGGCUAGGGGUGUACGCAUCAAGG----UUUU-----CCGAGAUGUUCAGCCCGCUAGUACUAAUAGUUCAAGAGAUAACUUAG

Tte GUAGAAGACGGGGUUGAUGGGGGGGCGGUGUAACCCCCGAGGG-UCUC----CCCGAGGGGG-GAGCCGGCCCCUCCCAAUCGCCCGAGCGUGCGGACGG

Sac AGAAGAGGGGGUUGAUGGGGUGGGGAUGUAAGCUCCAAGGU---UGUAAG--ACCGAGGAGUUUAGUCCGCCACUCCCAAUCAGGCUCCCCCCUGGUGGG

Mka AAAAGACCCGGUUGAUGGGGCGGGGGUGUAAGGCCCAAGGGGCCGUUG-GGCCCCGAGGGCCUGAGCCCACCGCUCCCAAUCGGCCGAUGGACCCGGCGG

Neq AGAAGACCCGGUUGAUGGGGGGGCGGUGGAAGCCCCGAGGG----UUU-----CCGAGGGGC-GAGCCGGCCCCUCCCAAUCGGGGGGUGGUCCUGCGGA

Iho AGAAGACGGGGUUGAUGGGGCGGGGGUGUAAGCCCCGAGGGG---UUU----CCCGAGGGGC-GAGCCCGCCGCUCCCAAUCGCCCGAGGCCGUUAGCCU

CON agAagAcggGguugauggGgggGgGguGuaagcccCaaGgg---uuUu----cCCGAGgggc-gAGcCcgCcccUcCcAAUcgcccgagcguccgggcgg
