# Supplementary material for: C/D box sRNA-guided 2′-O-methylation patterns of archaeal rRNA molecules
Source: BMC Genomics. 2015 Aug 22;16:632. doi: 10.1186/s12864-015-1839-z (PMC4644070; doi:10.1186/s12864-015-1839-z)
Supplement: Additonal file 4: — Methylation predictions in 16S rRNA alignment. The 16S rRNA sequences from the seven archaeal species used in this study were aligned using Infernal with manual adjustments; a consensus sequence (CON) is given at the bottom. The 16S rRNA sequence from Escherichia coli was included in the alignment for reference with positions numbered separately. Highlights and abbreviations are as listed in the legend to Additional file 3. (DOCX 57 kb) [file 12864_2015_1839_MOESM4_ESM.docx]

**16S rRNA alignment**

10 20 30 40 50 60 80 110

Eco GAAGAGUUUGAUCAUGGCUCAGAUUGAACGCUGGCGGCA—GGCCUAACACAUGCAAGUCGAACGG..AAGCAGCUUGCU....AGUGGCGGACGGGUGA

1 10 20 30 40 50 60 70 80 90 100

P19 P116 T4 P18P53T15 P116P19 P18S1T49 T49

K31 K23 K31I92 K23I206* I92 I129 I148K83

Pca AAACCGGUUGAUCCUGCCGGACCCGACCGCUAUCGGGGUAGGGCUAAGCCAUGCGAGUCGCGCGCCCGGGGGCGCCCGGG----AGCGGCGCACGGCUCA

Mma AUUCCGGUUGAUCCCGCCGGAGGCCACUGCUAUUGGGAUUCGACUAAGCCAUGCGAGUCU-AUGGUUUCGGC------------CAUGGCGGACGGCUCA

Tte AAACCGGUUGAUCCUGCCGGACCUGACCGCUAUCGGGGUGGGGCUAAGCCAUGCGAGUCGCGCGCCCGGGGCGCCGGG------CGCGGCGCACGGCUCA

Sac AUUCCGGUUGAUCCUGCCGGACCCGACCGCUAUCGGGGUAGGGAUAAGCCAUGGGAGUCUUACACUCCCGGGUAAGGGAG----UGUGGCGGACGGCUGA

Mka ACUCCGGUUGAUCCUGCCGGAGGCCACCGCUAUCGGGGUCCGACUAAGCCAUGCAAGUCGAGGGCCGCCCGGCAAUGGGGGCGGCCCGGCGGACGGCUCA

Neq ACUCCCGUUGAUCCUGCGGGAGGCCACCGCUAUCUCCGUCCGGCUAACCCAUGGAAGGCGAGGGUCCCCGGGUAAGGGGG----CCCGCCGCACGGCUGA

Iho ACUCCGGUUGAUCCUGCCGGACCCGACCGCUAUCGGGGUAAGGCUAAGCCAUGGGAGUCGAACGCCCGCCGCCGCGGG------CGUGGCGGACGGCUGA

CON AcUCCgGUUGAUCCUGCcGGAcccgACcGCUAUcggggUacGgcUAAgCCAUGcgAGUCgagcgcccccgGgcaaggGgG----cgcGgCGgACGGCUcA

120 130 140 150 160 170 180 190

Eco GUAAUGUCUGGG-AAGCUGCCUGAUGGAGGGGGAUAACUAC-UGGAAACGGUAGCUAAUACCGCAUAA-----------------CGUCGCAAGACCAAA

| | | | | | | | | 200

P100 M1N3 P100

K83 I414 K39/119 K83 I414I148 K39/119 K25K53K24 K14 K36

Pca GUAACACGUGCCUAACCUAACCUCGGGAGGGGGACACCCCC-GGGAAACUGGGGUCAAUCCCCCAUAGGGGAAGGGCGCUGGAAGGCCCCUUCCCCGAAA

Mma UUAACACGUGGUUAACAUACCCUCAGGUGGGGGAUAACCUU-GGGAAACUGAGGAUAAUACCCCAUAGAAAAAGCAGUCUGGAAUGAUUCUUUUUCGAAA

Tte GUAACACGUACCCAACCUAACCUCGGGAGGGGGACAACCCC-GGGAAACUGGGGCUGAUCCCCCAUAGGGGAAGGGCGCUGGAAGGCCCCUUCCUCCAAA

Sac GUAACACGUGGCUAACCUACCCUCGGGACGGGGAUAACCCC-GGGAAACUGGGGAUAAUCCCCGAUAGGGAAGGAGUCCUGGAAUGGUUCCUUCCCUAAA

Mka GUAACACGUGGGUAACCUACCCUCGGGACGGGGAUAACCCCGGCGAAAGUGGGGCUAAUCCCCGAUAGGCGGGGCGGCCUGGAACGGUCCUCCGCCGAAA

Neq GUAACACGUCGGUAACCUACCCUCGGGACGGGGAUAACCCC-GGGAAACUGGGGCUAAUCCCCGAUAGGGGAUGGGUGCUGGAAGGCCCCAUCCCCGAGA

Iho GUAACACGUGGCUAACCUACCCUCGGGAGGGGGAUAACACC-GGGAAACUGGUGCUAAUCCCCCAUAGGGGCGGAGGCCUGGAAGGGUUCCGCCCCGAAA

CON gUAACACGUggcUAACcUAcCCUCgGGagGGGGAUAaCccc-GgGAAAcUGggGcUaAUcCCCcAUAGgggaaGgggcCUGGAAgGcUcCUUcccCgAaA

200 210 220 230 240 250 260 270 280 290

Eco GAG----GGGGACCUUCGGGCCUC---UUGCCAUCGGAUGUGCCCAGAUGGGAUUAGCUUGUUGGUGGGGUAACGGCUCACCUAGGCGACGAUCCCUAGC

| | | | | | | | | 300

P20T28

I402 K75 K14 K24/25 K113 I83

Pca GGGCUGGCGGCCGAUCCGCCGCCAG--UCGCCCGAGGGUGGGGGCACGGCCCAUCAGGUAGUUGGCGGGUUAAAGGCCGGCCAAGCCGAAGACGGGUAGG

Mma -GCACAU--------------------GCGCCUGAGGAUUGGACUGCGCUCGAUUAGGUAGUUGGUGGGGUAAUGGCCCACCAAGCCUACGAUCGGUACG

Tte GGGAUCGCGGGCGAUCUCCCGCGGU--CCGCCCGAGGGUGGGGGUACGGCCCAUCAGGUUGUUGGCGGGGUAACGGCCCGCCAAGCCGAAGACGGGUAGG

Sac GGGCUAUAGGCUAUUUCCCGUUUGUAGCCGCCCGAGGAUGGGGCUACGGCCCAUCAGGCUGUCGGUGGGGUAAAGGCCCACCGAACCUAUAACGGGUAGG

Mka -GGGCCCGGGCCCAUGCCGCCCGGGU-CCGCCCGAGGAUGGGCCUGCGGCCGAUUAGGUAGUUGGCGGGGUAACGGCCCGCCAAGCCGAUAAUCGGUACG

Neq GGGGCUAGCGGUACUUCCCCCGCUAGCCCGCCCGAGGAUGGGCCGGCGGCCCAUCAGGUAGUUGGCGGGGUAAUGGCCCGCCAAGCCGAAGACGGGUAGG

Iho GGGGCUCGGGGGGGAACGCCCCGAGU-CCGCCCGAGGAUGGGGCCGCGCCCCAUCAGGUAGUUGGCGGGGUAAUGGCCCGCCAAGCCGAAGACGGGUAGG

CON GGggcacggGccgaUcccccccgggU-cCGCCcGAGGaUgGGgcUgCGgcCcAUcAGGUaGUUGGcGGGgUAAUGGCCcgCCaAgCCgAagAcgGGUAgG

300 310 320 330 340 350 360 370 380

Eco UGGUCUGAGAGGAU-GACCAGCCACACUGGAACUGAGACACGGUCCAGACUCCUACGGGAGGCAGCAGUGGGGAAUAUUGCACAAUGGGCGCAAGCCUGA

| | | | | | | | | 400

P31 N8 N8 T2

I121K21 K113 I121 I39K21

Pca GGCGGUGAGAGCCGCGAGCCCCGAGAUGGGCACUGAGACAAGGGCCCAGGCCCUACGGGGUGCAGCAGGCGCGAAUACUCCGCAAUGCGGGCAACCGCGA

Mma GGCCUUGAGAGAGG-GAGCCCGGAGAUGGGGACUGAGACACGGCCCCAGGCCcUACGGGGCGCAGCAGGCGCGAAACCUCCGCAAUGCACGAAAGUGCGA

Tte GGCGGUGAGAGCCGUGAGCCCCGAGAUGGGCACUGAGACAAGGGCCCAGGCCcUACGGGGUGCAGCAGGCGCGAAUACUCCGCAAUGCGGGCAACCGCGA

Sac GGCCGUGGAAGCGG-GAGCCUCCAGUUGGGCACUGAGACAAGGGCCCAGGCCcUACGGGGCGCACCAGGCGCGAAACGUCCCCAAUGCGCGAAAGCGUGA

Mka GGCGGUGAGAGCCG-GAGCCCGGAGACGGGGACUGAGACAAGGCCCCGGGCCcUACGGGGCGCAGCAGGCGCGAAACCUCCGCAAUGCGGGCAACCGCGA

Neq GGCCGUGAGAGCGG-GAGCCCCCAGAUCGGCACUGAGACAAGGGCCGAGGCCcUACGGGGCGCACCAGGGGCGAAACCUCCGCAAUGCGGGAAACCGUGA

Iho GGCCGUGGGAGCGG-GAGCCCCCAGAUGGGCACUGAGACAAGGGCCCAGGCCCUACGGGGCGCACCAGGCGCGAAAACUCCGCAAUGCGGGCAACCGUGA

CON GGCcgUGagAGcgG-GAGCCccgAGaUgGGcACUGAGACAaGGgCCcaGGCCCUACGGGGcGCAgCAGGcGCGAAaccUCCgCAAUGCggGcAAccGcGA

390 400 430 440 460 490 500 510 520

Eco UGCAGCCAUGCCGCGUGUAU----...GUAAAGU--.....ACUUUCAGCGGG..UAAAG..CCCGCAGAAGAAGCACC-GGCUAACUCCG-UGCCAGCA

| | | | | | | | | 500

T2 P7 P28T14S115 T5

K19K20 K19 K20 K1 K127 K28

I39 I104 I165I473 I166 I206 I152

Pca CGGGGCUACCCCGAGUGCCG----GGCGAAGAGCCC----GGCUUUUGCCCGGUCUAAAAAGCCGGGAGAAUAAG-CGGGGGGCAAGUCUGGUGUCAGCC

Mma CGGGGGGAUCCCAAGUGCUCA---UGCACA--GCAUG---GGCUUUUAUCAAGUGUAAACAGCUUGAGGAA-UAAGGGCUGGGCAAGUUCGGUGCCAGCA

Tte CGGGGCCACCCCGAGUGCCG----GGCGAAGAGCCC----GGCUUUUGCCCGGUGUAAGGAGCCGGGCGAA-UAAGCGGGGGGUAAGUCUGGUGUCAGCC

Sac GGGCGCUACCCCGAGUGCC-----UCCGCAA-GGA-----GGCUUUUCCCCGCUCUAAAAAGGCGGGGGAA-UAAGCGGGGGGCAAGUCUGGUGUCAGCC

Mka CGGGGGGACCCCGAGUGCCGUCGGGGCAAA--GCCCCGGCGGCUGUACCGGGGUGUAAAAAGCCCCGGGUAGAAAGCGGCGGGCAAGACCGCUGCCAGCC

Neq CGGGGGGACGGAGAGUGCCGGAG-GGCGUUAUGCUCUCC-GGCUUUUGGGGAGUGUAAGUAGCUCCCCGAA-UAAGCGGUGGGCAAGAGGGGUGGCAGCC

Iho CGGGGUUACCCCGAGUGCC-----CCCUCU-----CCGGGGGCUUUUCCCCGCUGUAAACAGGCGGGGGUAAUAAGCGGGGGGCAAGUCUGGUGUCAGCC

CON cGGgGcgAccccgAGUGCcg----ggCgaaa-Gcccc---GGCUUUUccccggUgUAAaaAGccggggGaA-UAaGcGggGGGcAAGUcUGgUGUCAGCc

530 540 550 560 570 580 590 600 610 620

Eco GCCGCGGUAAUACGGAGGGUGCAAGCGUUAAUCGGAAUUACUGGGCGUAAAGCGCACGCAGGCGGUUUGUUAAGUCAGAUGUGAAAUCCCCGGGCUCAAC

| | | | | | | | | 600

P6 P53T15 P28T14S115 P6T5 P53T15 T46?

K127 K105 K28 K4 K12 K105 K12

I206 I104I21 I166I165 I152 I64I34 I110 I64

Pca GCCGCGGUAAUACCAGCUCCGCGAGUGGUCGGGGUGUUUACUGGGCUUAAAGCGCCCGUAGCCGGCCCGGCAAGUCGCUCCUGAAAUCCCCGGGCUCAAC

Mma GCCGCGGUAACACCGACGGCCCGAGUGGUGGCCACUCUUAUUGGGCCUAAAGCGUCCGUAGCCGGUCCAGUAAGUCCCUGUUUAAAUCCUGCGGCUUAAC

Tte GCCGCGGUAAUACCAGCCCCGCGAGUGGUCAGGGUGAUUACUGGGCUUAAAGCGCCCGUAGCCGGCCCGGCAAGUCGCUCCUGAAAUCCCCAGGCUCAAC

Sac GCCGCGGUAAUACCAGCUCCGCGAGUGGUCGGGGUGAUUACUGGGCCUAAAGCGCCUGUAGCCGGCCCACCAAGUCGCCCCUUAAAGUCCCCGGCUCAAC

Mka GCCGCGGUAAUAGCGGCGCCGCAAGUGGUGGCCGCUUUUAUUGGGCCUAAAGGGGCCGUAGCCGGUCCCGUGGGUCCCCGCCGAAAGCCCGCGGCUUAAC

Neq GCCGCGGGAACACCCCCACCGCGAGCGGUGGCCGUGAUUAUUGGGCCUAAAGGGGCCGUAGCCGGGCCGGUGUGGCUCCGGUGAAAUCCUCGGGCUCAAC

Iho GCCGCGGUAAUACCAGCCCCGCGAGUGGUCGGGACGAUUAUUGGGCCUAAAGCGCCCGUAGCCGGCCUGGUGGCCCCCCUCCUAAAGCCCCGGGCUCAAC

CON GCCGCGGUAAUAcCagCccCgCgAGUGGUcggggUgaUUAUUGGGCcUAAAGcGcCcGUAGCCGGcCcggUaagUCcCcccUgAAAUcCcccGGCUcAAC

630 640 650 660 670 680 690 700 710 720

Eco CUGGGAAC-UGCAUCUGAUACUGGCAAGCUUGAGUCUCGUAGAGGGGGGUAGAAUUCCAGGUGUAGCGGUGAAAUGCGUAGAGAUCUGGAGGAAUACCGG

| | | | | | | | | 700

P136 P20T28 T28S19P20

I34K96I110K55I82I76I12 I437 K41/55/92I12I83K44I55 I314 I437 K4 K10/41I83K44

Pca CCGGGGGC-CGGGGGCGAUACUGCCGGGCUAGGGGGCGGGAGAGGCCGCCGGUACUCCGGGGGUAGGGGCGAAAUCCGUUAAUCCCCGGAGGACCACCAG

Mma CGCAGGAC-UGGCAGGGAUACUGCUGGACUUGGGACCGGGAGAGGACAAGGGUACUUCAGGGGUAGCGGUGAAAUGUGUUGAUCCUUGAAGGACCACCUA

Tte CUGGGGGC-AGGGGGCGAUACUGCCGGGCUAGGGGGCGGGAGAGGCCGCCGGUACUCCGGGGGUAGGGGCGAAAUCCUAUAAUCCCCGGAGGACCACCAG

Sac CGGGGAAC-UGGGGGCGAUACUGGUGGGCUAGGGGGCGGGAGAGGCGGGGGGUACUCCCGGAGUAGGGGCGAAAUCCUUAGAUACCGGGAGGACCACCAG

Mka CGCGGGAGUCGGCGGGGAAACUGCGGGACUUGGGACCGGGAGAGGCCGGAGGUACCCCCGGGGUAGGGGUGAAAUCCUGUCAUCCCGGGGGGACCGCCAG

Neq CCGAGGGCgCGCCGGAGCUACUACCGGCCUAGGGACCGGGAGGGGCCGACCGUACUCCCGGGGGAGCGGUGAAAUGCUGUAAUCCCGGGAGGACGACCCG

Iho CCGGGGAC-UGGAGGGGGUAGCGCCAGGCUAGGGGGCGGGAGAGGCCGAGGGUACUCCCGGGGUAGGGGCGAAAUCCGAUAAUCCCGGGAGGACCGCCAG

CON CcggGgac-cGgcgGcGaUAcUgccgGgCUaGGGggCGGGAGaGGccgacgGUACUcCcGGgGUAGgGGcGAAAUccUUUaAUcCcgGgaGGACcaCCag

730 740 750 760 770 780 790 800 810 820

Eco UGGCGAAGGCGGCCCCCUGGACGAAGACUGACGCUCAGGUGCGAAAGCGUGGGGAGCAAACAGGAUUAGAUACCCUGGUAGUCCACGCCGUAAACGAUGU

| | | | | | | | | 800

N11

K54 K60I26K10 I5I26 I55 K60 I5 K76

Pca UGGCGAAAGCGGGCGGCCAGAACGCGCCCGACGGUGAGGGGCGAAAGCCGGGGGAGCAAAGGGGAUUAGAUACCCCUGUAGUCCCGGCCGUAAACGAUGC

Mma UGGCGAAGGCACUUGUCUGGAACGGGUCCGACGGUGAGGGACGAAAGCCAGGGGCGCGAACCGGAUUAGAUACCCGGGUAGUCCUGGCCGUAAACUCUGC

Tte UGGCGAAAGCGGGCGGCCAGAACGCGCCCGACGGUGAGGGGCGAAAGCCGGGGGAGCAAAGGGGAUUAGAUACCCCUGUAGUCCCGGCCGUAAACGAUGC

Sac UGGCGGAAGCGCCCCGCUAGAACGCGCCCGACGGUGAGAGGCGAAAGCCGGGGCAGCAAACGGGAUUAGAUACCCCGGUAGUCCCGGCUGUAAACGAUGC

Mka UGGCGAAGGCGUCCGGCUGGAACGGGUCCGACGGUGAGGGCCGAAAGCCGGGGGAGCAAACCGGAUUAGAUACCCGGGUAGUCCCGGCUGUAAACGAUGC

Neq UGGCGAAAGCGGUCGGCCAGAACGGGUCCGACGGUGAGGGCCGAAGGCCGGGGGCUAGAACGGGAUUAGAGACCCCGGUAUUCCCGGCUGUCAACGCUGC

Iho UGGCGAAGGCGCUCGGCUGGAACGCGCCCGACGGUGAGGGGCGAAAGCCGGGGGAGCAAACCGGAUUAGAUACCCGGGUAGUCCCGGCUGUAAACGAUGC

CON UGGCGaAaGCgcUcggCUaGAACGcGcCCGACGGUGAGgGgCGAAaGCCgGGGgagcaAAcgGGAUUAGAUACCCcgGUAgUCCcGGCUGUaAACgaUGC

830 840 850 860 870 880 890 900 910

Eco CGACUUGGAG--GUUGUGCCCUUGAGGCGUGGC--UUCCGGAGCUAACGCGUUAAGUCGACCGCCUGGGGAGUACGGCCGCAAGGUUAAAACUCAAAUGA
 | | | | | | | | | 900

T19 T19 S101 S105 N25 P56T46S101S105P57

K48 K22 K91K32K3 K70 K22 K76 K32 K114 K70

I70 I70 I9 I409I203 I402 I203 I409I9

Pca GGGCUAGCUGUCGGUCGGGCUUAGGGCCCGGCCGGUGGCGAAGGGAAACCGUUAAGCCCGCCGCCUGGGGAGUACGGCCGCAAGGCUGAAACUUAAAGGA

Mma GAACUAGGUGUUAGGUAGGCCCCGUGCCUAUCUAGUGCCGAAGGGAAGCCGUUAAGUUCGCCGCCUGGGGAGUACGGUCGCAAGACUGAAACUUAAAGGA

Tte GGGCUAGCUGUCGGCCGGGCUUAGGGCCCGGCCGGUGGCGUAGGGAAACCGUUAAGCCCGCCGCCUGGGGAGUACGGCCGCAAGGCUGAAACUUAAAGGA

Sac GGGCUAGGUGUCGAGUAGGCUUAGAGCCUACUCGGUGCCGCAGGGAAGCCGUUAAGCCCGCCGCCUGGGGAGUACGGUCGCAAGACUGAAACUUAAAGGA

Mka GGACUAGGUGUUGGGGCGGCCACGAGCCGCCCCAGUGCCGUAGGGAAGCCGUUAAGUCCGCCGCCUGGGGAGUACGGCCGCAAGGCUGAAACUUAAAGGA

Neq GGGCUACCUGCUGGGCGGGCUACGAGCCCGCCCAGUGGGGUAGGGAAGCCGUUAAGCCCGCCGCCUGGGGAGUACGGCCGCAAGGCUGAAACUUAAAGGA

Iho GGGCUAGGUGUUGGGCGGGCUUCGAGCCCGCCCAGUGCCGCAGGGAAGCCGUUAAGCCCGCCGCCUGGGGAGUACGGCCGCAAGGCUGAAACUUAAAGGA

CON GggCUAggUGUUgggcgGGCUUcGaGCCcgcccaGUGccGUAGGGAAgCCGUUAAGccCGCCGCCUGGGGAGUACGGcCGCAAGgCUGAAACUUAAAGGA

920 930 940 950 960 970 980 1000 1010

Eco AUUGACGGGGGCCCGC-ACAAGCGGUGGAGCAUGUGGUUUAAUUCGAUGCAACGCGAAGAACCUUACCUGG...GACAUCCACG-GAA..UCAGAGAUGA

| | | | | | | | | 1000

N17 P57T46P56 N15 N25 P47

I402 I55 K62 K93

Pca AUUGGCGGGGGGGCACCACAAGGGGUGAAGCUUGCGGCUUAAUUGGAGUCAACGCCGGAAACCUCACCCGGGGCGACAGCAGGAUGAAGGCCAGGCUAAC

Mma AUUGGCGGGGGAGCACCACAACGGGUGGAGCCUGCGGUUUAAUUGGAUUCAACGCCGGGCAUCUCACCAGGAGCGACAGCAUGAUGACGGCCAGGUUGAC

Tte AUUGGCGGGGGGGCACCACAAGGGGUGAAGCUUGCGGCUUAAUUGGAGUCAACGCCGGAAACCUUACCCGGGGCGACAGCAGGAUGAAGGCCAGGCUAAC

Sac AUUGGCGGGGGAGCACCACAAGGGGUGGAACCUGCGGCUCAAUUGGAGUCAACGCCUGGAAUCUUACCGGGGGAGACCGCAGUAUGACGGCCAGGCUAAC

Mka AUUGGCGGGGGAGCACCACAACCGGUGGAGCCUGCGGUUUAAUUGGAUUCAACGCCGGAAACCUUACCGGGGGCGACAGCAGGAUGAAGGCCAGGUUGAC

Neq AUAGGCGGGGGAGCAC-ACAAGAGGUGGGGUGCGCGGUUUAAUUGGAUUCGACGCCGGGAACCUCACCGGGGCUGACAGCACAAUGAUGGUCGGCCUGAA

Iho AUUGGCGGGGGAGCACCACAAGGGGUGGAGCCUGCGGCUUAAUUGGAGUCAACGCCGGGAACCUUACCGGGGGCGACAGCAGGAUGAAGGUCAGGCUGAA

CON AUUGGCGGGGGaGCACCACAAggGGUGgagccUGCGGcUUAAUUGGAgUCaACGCCgGgaAcCUUACCgGGggcGACaGCAggAUGAaGGcCaGgcUgAc

1020 1030 1050 1060 1070 1080 1090 1100 1110 1120

Eco G.CCUU..GGGAACCG..GACAGGUGCUGCAUGGCUGUCGUCAGCUCGUGUUGUGAAAUGUUGGGUUAAGUCCCGCAACGAGCGCAACCCUUAUCCUUUG

| | | | | | | | | 1100

P49S6 S5P3T12P47 P49 T12 S5 P100 S6*

I310 K124 K120I310 I147K124I103K62 I147 K93 K122K79

Pca GACCUUGCCGGACGAGCUGAGAGGAGGUGCAUGGCCGUCGUCAGCUCGUGCCGUGAGGUGUCCGGUUAAGUCCGGCAACGAGCGAGACCCCCGCCCCUAG

Mma GACCUUGCCUGAAGCGCUGAGAGGUGGUGCAUGGCCAUCGUCAGCUCGUACCGCGAGGCGUCCUGUUAAGUCAGGUAACGAGCGAGACCCGUGCCCUAUG
Tte GACCUUGCCGGACGAGCUGAGAGGAGGUGCAUGGCCGUCGUCAGCUCGUGCCGUGAGGUGUCCGGUUAAGUCCGGCAACGAGCGAGACCCCCACCCCUAG

Sac GACCUUGCCUGACUCGCGGAGAGGAGGUGCAUGGCCGUCGCCAGCUCGUGUUGUGAAAUGUCCGGUUAAGUCCGGCAACGAGCGAGACCCCCACCCCUAG

Mka GACCUUGCCGGACGAGCUGAGAGGAGGUGCAUGGCCGCCGUCAGCUCGUGCCGUGAGGUGUCCUGUUAAGUCAGGUAACGAGCGAGACCCCCGCCCGCAG

Neq GGGCCUACCGGAGGCGCUGAGAGGAGGUGCAUGGCCGCCGUCAGCCUGUGCCGUGAGGUGCCCUGUUAAGUCAGGAAACAGGCGAGACCCGCGCCCGCAG

Iho GACCUUACCUGACGCGCUGAGAGGAGGUGCAUGGCCGUCGCCAGCUCGUGCCGUGAGGUGUCCGGUUAAGUCCGGCAACGAGCGAGACCCCCGUCCCCAG

CON GacCUUgCCgGAcgcGCUGAGAGGaGGUGCAUGGCCgUCGUCAGCUcGUgccGUGAggUGUCCgGUUAAGUCcGGcAACgaGCGAGACCCccgcCCccaG

1130 1140 1150 1160 1170 1180 1190 1200 1210

Eco UUGCCAGCGG----UCCG------GCCGGGAACUCAAAGGAGACUGCCAGUGA-UAAACUGGAGGAAGGUGGGGAUGACGUCAAGUCAUCAUGGCCCUUA

| | | | | | | | | 1200

P16 P120 P100 P120 P23 P32 P31T34

K100 K96 K74 K122 I104K52I103K96 I412 K100 I412 K52 I472

Pca UUGCUACCCCUCCCUACG--GGGAGGGGGGCACACUAGGGGGACUGCCGGCG--UAAGCCGGAGGAAGGAGGGGGCCACGGCAGGUCAGUAUGCCCCGAA

Mma UUGCUACUUUUUCCUCCG--GGAG-AAAGGCACUCAUAGGGGACCGCUGGCGC-UAAGUCAGAGGAAGGAGCGGGCAACGAUAGGUCCGCAUGCCCCGAA

Tte UUGCUACCCCGCUCUUCG--GGGCGGGGGGCACACUAGGGGGACUGCCGGCG--UAAGCCGGAGGAAGGAGGGGGCGACGGCAGGUCAGUAUGCCCCGAA

Sac UUGGUAUUCUGGACUCCG--GUCC-AGAACCACACUAGGGGGACUGCCGGCG--UAAGCCGGAGGAAGGAGGGGGCCACGGCAGGUCAGCAUGCCCCGAA

Mka UUGCCAGCGGGCCCCGUAAGGGGCGCCGGGCACUCUGCGGGGAUCGCCGCCGU-UAAGGCGGAUGAAAGUGGGGGCGACGGCAGGUCCGUAUGCCCCGAA

Neq UUGCGACGG---CCGAAA--GG----CCGGCACACUGCGGGGACUGCCGGGG--AAACCCGGAGGAAGGUGCGGGCGACGGCAGGUAUGCAUGCCCCGAA

Iho UUGCUACCCGGGGCUCCG---GCCCCGGGGCACACUGGGGAGACUGCCGCCGUAUAAGGCGGAGGAAGGAGGGGGCUAUGGCAGGUCAGCAUGCCCCGAA

CON UUGcUAccccgccCUccg--GggcgaggggCACaCUagGGgGAcUGCcGgcG--UAAgcCgGAgGAAgGaGgGGGCgAcGgcAGGUcaGcAUGCCCCGAA

1220 1230 1240 1250 1260 1270 1280 1290 1300 1310

Eco CGACCAGGGCUACACACGUGCUACAAUGGCGCAUACAAAGAGAAGCGACCUCGCGAGAGCAAGCGGACCUCAUAAAGUGCGUCGUAGUCCGGAUUGGAGU

| | | | | | | | | 1300

P32 T20 P31T34N15P23 P135 P135 T20P4S112

I417 I11 K30 I422 K30 I472 K68 I417

Pca ACCCCGGGGCUGCACGCGAGCUGCAAUGGCGGGGACAGCGGGAUCCGACCCCGAAAGGGGGAGGCAAUCCCGUAAACCCCGCCCCAGUAGGGAUCGAGGG

Mma UCUCCUGGGCUACACGCGGGCUACAAUGGCUAGGACAAUGGGCCGCAACCCUGAAAAGGGACGCAAAUCUCCUAAACCUAGUCGUAGUUCGGAUCGUGGG

Tte ACCCCGGGGCUGCACGCGAGCUGCAAUGGCGGGGACAGCGGGAUCCGACCCCGAAAGGGGGAGGCAAUCCCGUAAACCCCGCCCCAGUAGGGAUCGAGGG

Sac ACUCCCGGGCCGCACGCGGGUUACAAUGGCAGGGACAACGGGAUGCUACCUCGAAAGGGGGAGCCAAUCCUU-AAACCCUGCCGCAGUUGGGAUCGAGGG

Mka ACCCCCGGGCUACACGCGGGCUACAAUGGCGGGGACAAUGGGAUCCGACCCCGAAAGGGGGAGGAAAUCCCCUAAACCCCGUCGUAGUUCGGAUUGCGGG

Neq UGCCCCGGGCUACACGCGCGCAUCAAUGGGCGGGACAGGGGGCCGCGACCCCGAAAGGGGGAGCAAAUCCCC-AAACCCGCUCUCAGUCCAGAUCGAGGG

Iho ACCCCCGGGCUGCACGCGGGCUACAAUGGCGGGGACAGCGGGUUGCGACCCCGAAAGGGGGAGCCAAUCCCUGAAACCCCGCCGAGGUUGGGAUCGAGGG

CON accCCcGGGCUgCACGCGgGcUaCAAUGGcggGGACAgcGGGaUgCgACCccGAAAgGGGgaGccAAUCcccUAAACCccgcCgcaGUUggGAUcGaGGG

1320 1330 1340 1350 1360 1370 1380 1390 1400 1410

Eco CUGCAACUCGACUCCAUGAAGUCGGAAUCGCUAGUAAUCGUGGAUCAGAAUGCCACGGUGAAUACGUUCCCGGGCCUUGUACACACCGCCCGUCACACCA

| | | | | | | | | 1400

S118P4 S22 P111T21 P25T35 P56 S118 N17 P111T21 P25*T35* N13S129

K35 I422 K35 I81* I56 I81

Pca CUGCAACUCGCCCUCGUGAACGUGGAAUCCCUAGUAACCGCGUGUCACCAACGCGCGGUGAAUACGUCCCUGCCCCUUGCACACACCGCCCGUCGCACCA

Mma CUGUAACUCGCCCACGUGAAGCUGGAAUCCGUAGUAAUCGCAGUUCAUAAUACUGCGGUGAAUGUGUCCCUGCUCCUUGCACACACCGCCCGUCACACCA

Tte CUGCAACUCGCCCUCGUGAACGUGGAAUCCCUAGUAACCGCGUGUCACCAACGCGCGGUGAAUACGUCCCUGCCCCUUGCACACACCGCCCGUCGCACCA

Sac CUGAAACCCGCCCUCGUGAACGAGGAAUCCCUAGUAACCGCGGGUCAACAACCCGCGGUGAAUACGUCCCUGCUCCUUGCACACACCGCCCGUCGCUCCA

Mka CUGCAACUCGCCCGCAUGAAGGUGGAAUCGGUAGUAACCGUGCCUCAGAAUGGCACGGUGAAUACGUCCCUGCUCCUUGCACACACCGCCCGUCACGCCA

Neq CUGCAACUCGCCCUCGUGACGGCGGAAUCUCUAGUAGUCGGACGUCACCAGCGUCCGGCGAAUACGUCCCUGCUCCUUGCACUCACCGCCCGUCAAGCCA

Iho CUGCAACUCGCCCUCGUGAACGCGGAAUCCCUAGUAACCGCGCGUUAGCAUCGCGCGGUGAACACGUCCCUGCUCCUUGCACACACCGCCCGUCGCUCCA

CON CUGcAACUCGCCCUCgUGAacgUGGAAUCccUAGUAacCGcgcgUcAccAacgcgCGGUGAAUacGUCCCUGCUCCUUGCACaCACCGCCCGUCgcaCCA

1420 1430 1440 1470 1480 1490 1500 1510

Eco UGGGAGUGGGUUGCAAAAGAA------GUAGGUAG....................ACUUUGUGAUUCAUGACUGGGGUGAAGUCGUAACAAGGUAACCGU

| | | | | | | | | 1500

P118 M5

I56 K36

Pca CCCGAGGGAGCCCUCUGCGAGGCCCCUCGCCGCAA--------GGUGGGGGGACGAGCAGGGGGCUCCCAAGGGGGGUGAAGUCGUAACAAGGUAGCCGU

Mma CCCGAGUUGGGUUGAAGUGAGGCCUUGGCCUU-----------UGGCUAGGGUCGAACUUGGGCUCAGCGAGGGGGGUGAAGUCGUAACAAGGUAGCCGU

Tte CCCGAGGGAGUUCUCUGCGAGGCCCCUCGCUUGGGGCAACCCAGGUGGGGGGACGAGCAGAGAACUCCCGAGGGGGGUGAAGUCGUAACAAGGUAGCCGU

Sac CCCGAGCGAGAaAGGGGUGAGGUCCCUUGCGaUa---------AGUGGGGGAUCGAACUCCUUUCCCGCGAGGGGGGAGAAGUCGUAACAAGGUAGCCGU

Mka CCCGAGCCCCCCGGGGGCAAGCCCCCGGUC-CGCAA-------GGGCUGGGGGCGAGCCCCCGGGGGGUGAGGGGGGCGAAGUCGUAACAAGGUAGCCGU

Neq CCCGAGCUGGGGCCUAGCGAGGCCGUGGGGGGUUCGC------CCCCCACGGUCGAGCUAGGCCCCGGCGAGGGGGGCUAAGUCGACACAAGGUAGCCGU

Iho CCCG------------------------------------------------------------------AGGGGGGAGAAGUCGUAACAAGGUAGCCGU

CON CCCGAGcgagcccgcaGcgAGgcCccgggcgggaa--------ggUcgggGgUCGAgCUcgggccccgcgAGGGGGGUgAAGUCGUaACAAGGUAGCCGU

1520 1530 1540

Eco AGGGGAACCUGCGGUUGGAUCACCUCCUUA

| | | | | | | | | 1600

Pca AGGGGAACCUGCGGCUGGAUCACCUCCA

Mma AGGGGAACCUGCGGCUGGAUCACCUCCU

Tte AGGGGAACCUGCGGCUGGAUCACCUCCU

Sac AGGGGAACCUGCGGCUGGAUCACCUCAU

Mka AGGGGAACCUGCGGCUGGAUCACCUCCA

Neq AGGGGAACCUGCGGCUGGAUCACCUCCU

Iho AGGGGAACCUGCGGCUGGAUCACCUCCC

CON AGGGGAACCUGCGGCUGGAUCACCUCcU
